# B cell‐intrinsic changes with age do not impact antibody‐secreting cell formation but delay B cell participation in the germinal centre reaction

**DOI:** 10.1111/acel.13692

**Published:** 2022-08-18

**Authors:** Jia Le Lee, Sigrid C. Fra‐Bido, Alice R. Burton, Silvia Innocentin, Danika L. Hill, Michelle A. Linterman

**Affiliations:** ^1^ Immunology Program Babraham Institute Cambridge UK; ^2^ Department of Immunology and Pathology Monash University Melbourne Victoria Australia

**Keywords:** ageing, antibodies, B cells, vaccine response

## Abstract

Vaccines typically protect against (re)infections by generating pathogen‐neutralising antibodies. However, as we age, antibody‐secreting cell formation and vaccine‐induced antibody titres are reduced. Antibody‐secreting plasma cells differentiate from B cells either early post‐vaccination through the extrafollicular response or from the germinal centre (GC) reaction, which generates long‐lived antibody‐secreting cells. As the formation of both the extrafollicular antibody response and the GC requires the interaction of multiple cell types, the impaired antibody response in ageing could be caused by B cell intrinsic or extrinsic factors, or a combination of the two. Here, we show that B cells from older people do not have intrinsic defects in their proliferation and differentiation into antibody‐secreting cells *in vitro* compared to those from the younger donors. However, adoptive transfer of B cells from aged mice to young recipient mice showed that differentiation into extrafollicular plasma cells was favoured at the expense of B cells entering the GC during the early stages of GC formation. In contrast, by the peak of the GC response, GC B cells derived from the donor cells of aged mice had expanded to the same extent as those from the younger donors. This indicates that age‐related intrinsic B cell changes delay the GC response but are not responsible for the impaired antibody‐secreting response or smaller peak GC response in ageing. Collectively, this study shows that B cells from aged individuals are not intrinsically defective in responding to stimulation and becoming antibody‐secreting cells, implicating B cell‐extrinsic factors as the primary cause of age‐associated impairment in the humoral immunity.

## Graphical abstract

The effects of cell‐intrinsic changes on B cells function during ageing remain unknown. Here, we found that B cells from older humans do not have defects in activation, proliferation and antibody‐secreting cell differentiation when stimulated *in vitro*. B cells from aged mice displayed a delay in germinal centre formation and preferential differentiation into extrafollicular plasma cells, compared to B cells from young mice early post‐immunisation, but mounted comparable germinal centre when the germinal centre response has matured.

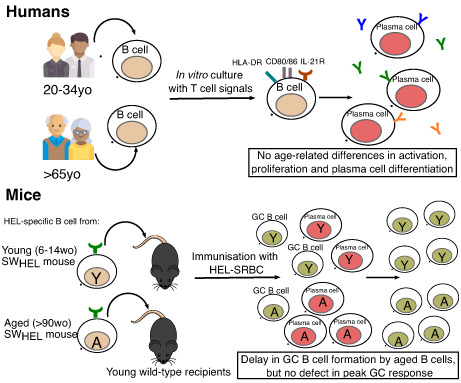

**Abbreviations**
AID: activation‐induced cytidine deaminase
DZ: dark zone
GC: germinal centre
HEL: hen egg lysozyme
HEL‐SRBC: HEL conjugated to sheep red blood cells
LZ: light zone
SWHEL: Switched‐HEL
WT: wild‐type

## 1 INTRODUCTION

Ageing is associated with a functional decline in the immune system, which results in increased susceptibility to infections and more severe disease outcomes among older individuals (Gavazzi & Krause, 2002; Weiskopf et al., 2009). Vaccines represent an important preventive intervention for protecting against (re)infections and minimising disease severity in vaccinated individuals by promoting the generation of pathogen‐specific antibodies. However, the formation of antibody‐secreting plasma cells and antigen‐specific antibodies is reduced in older individuals, contributing to limited vaccine efficacy to multiple vaccines in older individuals (Bums et al., 1993; Hainz et al., 2005). This age‐related defect in vaccine‐induced antibody responses was also observed more recently for COVID‐19 vaccines after one dose, though a second dose was found to be effective in boosting the responses in older people (Collier et al., 2021; Li et al., 2021; Ramasamy et al., 2020). Understanding the mechanisms underpinning the age‐related defects in vaccine‐induced antibody production is important to guide strategies aimed at improving vaccine efficacy among older people.

Vaccine‐induced antibodies are derived from B cells via two pathways: the extrafollicular response, which generates short‐lived plasma cells early after vaccination, or the GC reaction, which peaks later. To initiate these responses during T‐dependent vaccination, antigen‐activated B cells interact with primed T cells at the T cell–B cell border in secondary lymphoid organs. Here, signals provided by T cells (such as CD40L and IL‐21) promote B cell proliferation and differentiation towards the extrafollicular or GC fates (Lee et al., 2011; Weinstein et al., 2016). In the extrafollicular pathway, B cells migrate to the extrafollicular foci regions of the spleen or the lymph node medullary cords and differentiate into short‐lived plasmablasts (MacLennan et al., 2003). These plasmablasts provide an early wave of antibodies to control early infection, but do not confer long‐term immunity (Luther et al., 1997; Smith et al., 1996). Alternatively, B cells can enter the GC reaction, which are specialised microstructures formed in the follicular regions of secondary lymphoid tissues later in the immune response (Mesin et al., 2016). B cells undergo somatic hypermutation of their immunoglobulin genes followed by a selection process, which involves interaction with follicular dendritic cells and T follicular helper cells (Mesin et al., 2016). Eventually, positively selected GC B cells exit the GC as long‐lived antibody‐secreting plasma cells or memory B cells. Since these cells are long‐lived and have high affinity for antigen, they are crucial in conferring long‐lasting protection against infections post‐vaccination.

As the formation and maintenance of the extrafollicular response and GC reaction require the complex interactions of multiple different cell types (e.g., B cells, T cells, dendritic cells and stromal cells), the impaired humoral response during vaccination in older people could be caused by B cell‐intrinsic and/or extrinsic alterations. B cell‐intrinsic changes that might contribute to the age‐related decline in the humoral immune response have been reported. B cells from aged mice and humans were shown to have impaired class‐switch recombination due to reduced expression of the activation‐induced cytidine deaminase (AID), an enzyme required for the initiation of the class‐switch recombination process and somatic hypermutation, and its positive regulator, the E47 transcription factor (Frasca et al., 2004, 2008). Additionally, B cells from aged mice were reported to have reduced expansion following immunization in a young recipient environment, and this proliferative defect was also observed *in vitro* following anti‐CD40 and IL‐4 or LPS stimulation (Blaeser et al., 2008; Dailey et al., 2001).

In this study, we used both an *in vitro* culture systems of human and mouse B cells and *in vivo* mouse experiments where transgenic B cells from aged mice were adoptively transferred into younger recipients to determine whether cell‐intrinsic changes with age alter B cells' ability to respond to stimulation and differentiate into antibody‐secreting plasma cells. Both naïve and memory B cells from older people displayed comparable proliferation, activation and antibody‐secreting cell formation as B cells from young adults upon stimulation with T cells signals *in vitro*. Stimulated naïve and memory B cells from older donors also had a similar antibody‐secreting capacity to those from younger donors. To understand how B cell‐intrinsic ageing affects the immune response to immunisation *in vivo*, we used adoptive transfer of transgenic B cells. Six days after immunisation, B cells from aged mice preferentially differentiated into extrafollicular plasma cells and generated fewer GC B cells compared to B cells from the younger donors. However, 10 days after immunisation, the magnitude of the GC response was comparable between age groups, indicating that B cell‐intrinsic changes that occur in ageing likely delay the initiation of GC response but are insufficient to explain entirely the poor GC response observed in older individuals. Correspondingly, the transfer of HEL‐specific B cells from young mice into aged recipient mice resulted in a significant defect in the number of transferred B cells recovered as compared to B cells transferred into a young environment. Together, we report that B cells from older mice and humans generally do not have intrinsic defects in responding to stimulation and differentiating into plasma cells, but may contribute to the delayed kinetics of the GC response in ageing, implicating B cell‐extrinsic factors to be the primary contributors for the age‐related defects in humoral response post‐vaccination.

## 2 RESULTS

### 2.1 B cells from older people do not have defects in proliferation or differentiation into functional antibody‐secreting plasma cells

To assess if there are any age‐associated intrinsic defects in human B cells' ability to differentiate into plasma cells, an *in vitro* differentiation assay was established. B cells isolated from the peripheral blood of younger (20–34 years old) and older (68–76 years old) people were flow sorted into naïve and memory B cell subsets (gating strategy shown in Figure S1A). Naïve B cells used in cultures were sorted live CD20^+^ CD10^−^ CD27^−^ IgD^+^ cells while memory B cells consisted of the three subsets of memory cells (unswitched CD27^+^ IgD^+^, switched CD27^+^ IgD^−^ and atypical CD27^−^ IgD^−^). A fixed number (2.5 × 10^4^) of naïve and memory B cells from each donor was cultured and stimulated with CD40L and IL‐21 for 6 days, in order to gain an understanding of their functional capacity independent of changes in cell numbers with age. There was a significant decrease in the percentage of CD10^−^ CD20^+^ B cells in the peripheral blood of older donors compared to younger donors (Figure S1B), consistent with previous reports (Frasca et al., 2008; Frasca & Blomberg, 2009). However, no statistically significant differences were observed in the percentages of naïve and memory B cells between younger and older donors (Figure S1C). These findings were replicated in a larger independent cohort of 21 young adult (18–36 years old) and 19 older (66–98 years old) human volunteers (Figure S1D).

**FIGURE 1:**
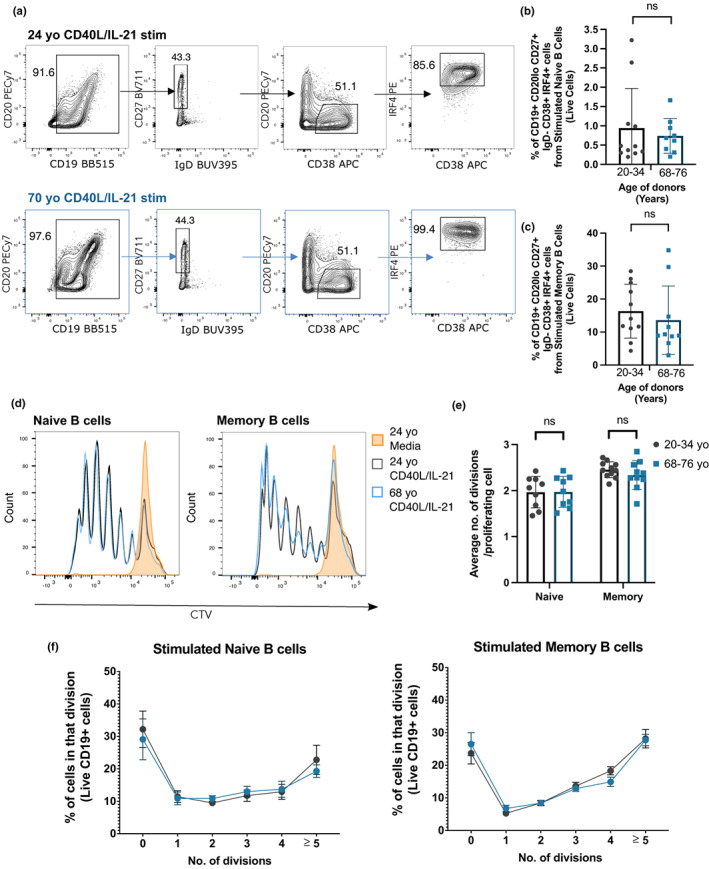
B cells from older human donors do not have defects in differentiating into plasma cells and proliferating upon stimulation. (a) Gating strategy for plasma cells (CD19^+^ CD27^+^ IgD^−^ CD20lo CD38^+^ IRF4^+^) 6 days after memory B cells were stimulated with CD40L and IL‐21 or unstimulated (cultured with media). Cells were pre‐gated for live cells and single cells. (b, c) Percentages of plasma cells out of live cells derived from naïve (b) and memory B cells (c) of younger (20–34 years old) and older donors (68–76 years old) after 6 days stimulation with CD40L and IL‐21. Data representative of four independent repeat experiments. (d) Representative flow cytometric histograms showing the cell trace violet stains of live naïve and memory CD19^+^ B cells from a 24 year old (grey) and 68 year old (blue) donor after 6 days incubation with CD40L and IL‐21 or media (orange). (e) Graph depicting the average number of divisions undergone by proliferating CD19^+^ naïve and memory B cells after 6 days stimulation with CD40L and IL‐21. (f) Graph showing the percentage of B cells in each division after 6 days stimulation with CD40L and IL‐21. Bar height corresponds to the mean, error bars indicate standard deviation, and each symbol represents values from independent donors. Statistics were calculated using the unpaired Mann–Whitney *U* test. Data were representative of three independent repeat experiments.

At day 6 post‐stimulation with CD40L and IL‐21, we did not observe any significant differences in the frequency of plasma cells (defined as CD19^+^ CD20lo CD27^+^ IgD^−^ CD38^+^ IRF4^+^ cells) derived from naïve and memory B cells from younger and older donors (Figure 1a–c). In addition, there was no age‐related difference in the proliferative capacity of the stimulated B cells (Figure 1d–f), as shown by the similar average number of cell divisions undergone by proliferating B cells (Figure 1e) and the percentage of cells in each division (Figure 1f). Consistent with no changes in plasma cell formation upon CD40L and IL‐21 stimulation, no significant age‐related differences in the expression levels of CD40 and IL‐21R, or the B cell activation marker CD38 was observed in naïve and memory B cells pre‐stimulation (Figure S2A–C). However, both naïve and memory B cells from older donors were observed to have lower expression levels of IRF4 than those from younger donors pre‐stimulation (Figure S2D). Together, our *in vitro* differentiation assay data shows that B cells from older donors do not have a defect in proliferating and differentiating into plasma cells after stimulation with CD40L and IL‐21.

**FIGURE 2:**
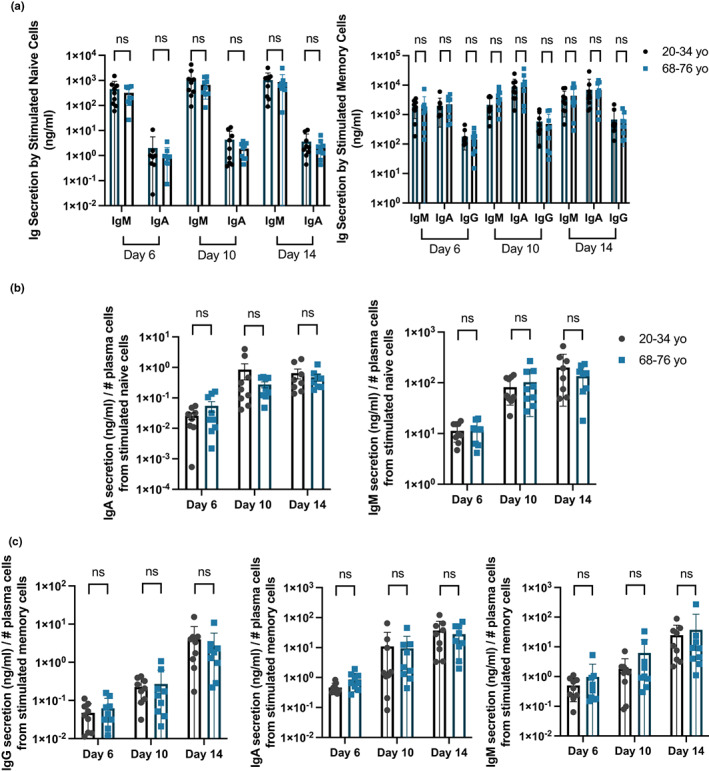
Stimulated naïve and memory B cells of older human donors have no defects in secreting antibodies. (a) Graphs showing the total amount of IgM, IgA and IgG secreted in the cultures of stimulated naïve (left) and memory (right) B cells 6, 10 and 14 days post‐stimulation with CD40L and IL‐21. (b, c) Graphs comparing the antibody‐secreting capacity (Ig concentration divided by number of antibody‐secreting cells) derived from stimulated (b) naive and (c) memory cells from younger and older donors after stimulation with CD40L and IL‐21. Number of antibody‐secreting cells was determined by flow cytometry with counting beads. Bar height corresponds to the mean, error bars indicate standard deviation, and each symbol represents values from independent donors. Statistics were calculated using the unpaired Mann–Whitney *U* test. Data were representative of three independent repeat experiments.

Next, we compared the antibody‐secreting capacity of stimulated naïve and memory B cells of young adult and older donors. Similar to what was previously reported (Deenick et al., 2013), IgM was principally detected in the supernatants of stimulated naïve B cells while IgG, IgA and IgM are detected in those of stimulated memory B cells (Figure 2a). We observed no significant differences in the concentration of antibodies detected in the cultures of stimulated naïve and memory B cells from younger and older donors, after 6, 10 and 14 days of culture (Figure 2a), consistent with comparable percentages and numbers of plasma cells in cultures derived from B cells from younger and older donors at each timepoint (Figure S3). To determine the antibody‐secreting capacity on a per‐cell basis, we normalised the Ig concentration with the number of antibody‐secreting cells in each well and observed that stimulated naïve and memory B cells from older donors have similar antibody‐secreting capacity as those from younger donors (Figure 2b,c).

To further probe the ability of B cell from older donors to respond to external stimuli, we stimulated naïve and memory B cells from younger and older donors using different combinations of T‐dependent and T‐independent signals, namely, CD40L/IL‐21/IL‐2, CpG, which is a Toll‐like receptor 9 agonist, and CD40L/CpG. Across all conditions, no age‐related defects was observed in plasma cell differentiation and proliferation (Figure S4A,B). IL‐21 was also shown to be key in promoting plasma cell differentiation as stimulating the B cells with CD40L alone or CD40L/IL‐2 yielded minimal differentiation, consistent with previous reports (Figure S4A) (Moens & Tangye, 2014). After 6 days of stimulation, we detected primarily IgM in supernatants from stimulated naïve B cells cultures and IgG, IgA and IgM in supernatants from stimulated memory B cells cultures (Deenick et al., 2013; Figure S4C–F). Only IgM and IgA were present in detectable levels in supernatants from memory B cells stimulated with CpG or CD40L/CpG (Figure S4D), similar to what was previously reported (Deenick et al., 2013). No significant defects in antibody‐secreting capacity were observed in naïve and memory B cells from older human donors across all stimulation conditions (Figure S4C‐F). Together, this demonstrates that B cells from older humans do not have intrinsic age‐related defects in differentiating into functional antibody‐secreting plasma cells, as compared to B cells from younger people.

### 2.2 B cells from older people are able to upregulate costimulatory molecules to a similar extent as those from young people

**FIGURE 3:**
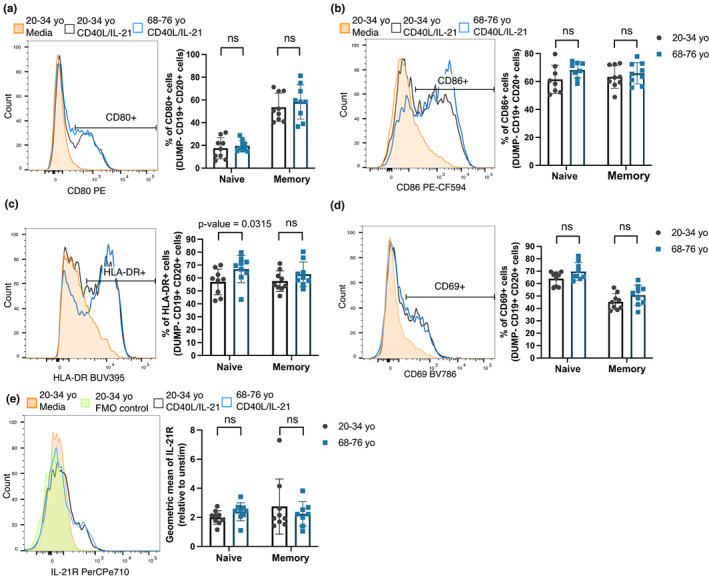
B cells from older human donors are able to upregulate costimulatory molecules and activation markers after stimulation. (a–e) Representative flow cytometric histograms showing the expression levels of costimulatory molecules by memory B cells from a 29 year old (grey) and 70 year old (blue) donor after 48 h stimulation with CD40L and IL‐21 or media (orange) and graphs showing the percentages of cells positive for the marker, or geometric mean of marker using the gating strategies shown, for CD80 (a), CD86 (b), HLA–DR (c), CD69 (d) and IL‐21R (e). These were gated on live CD19^+^ CD20^+^ cells. Fluorescent minus one (FMO) controls were included for the IL‐21R stain and are shown on the representative histogram. Bar height corresponds to the mean, error bars indicate standard deviation, and each symbol represents values from independent donors. Statistics were calculated using the unpaired Mann–Whitney *U* test. Data were representative of three independent repeat experiments.

Upon activation, B cells increase expression of CD80 and CD86 (ligands for CD28) and HLA–DR (ligand for T cell receptor), which are important for the activation of T cells and reciprocally, for B cells to receive survival and differentiation signals during T‐dependent responses (Attridge et al., 2014; Elgueta et al., 2009; Greenfield et al., 1998). After 48 h of stimulation with CD40L and IL‐21, stimulated naïve and memory B cells upregulated CD80, CD86 and HLA–DR, compared to their unstimulated counterparts, with no age‐dependent differences in expression generally (Figure 3a–c). Consistent with this observation, previous studies have reported no age‐related differences in the *ex vivo* expression of CD80, CD86 and CD40 on B cells from younger and older people, suggesting no B cell‐intrinsic defects in these activation pathways (Colonna‐Romano et al., 2003; Frasca et al., 2011). A significant increase in HLA–DR expression on stimulated naïve B cells from older donors, as compared to those from younger donors, was observed, but not for memory cells (Figure 3c). There was also no significant differences in the expression levels of CD69 and IL‐21R, which are upregulated during B cell activation, between stimulated naïve and memory B cells from younger and older donors (Figure 3d,e; Good et al., 2006; Xu et al., 2000). Collectively, the data from our human *in vitro* studies shows that there are no B‐cell intrinsic defects with age in activation after stimulation and their subsequent differentiation into plasma cells.

### 2.3 B cells from aged mice do not have defects in proliferation *in vivo* after immunisation

**FIGURE 4:**
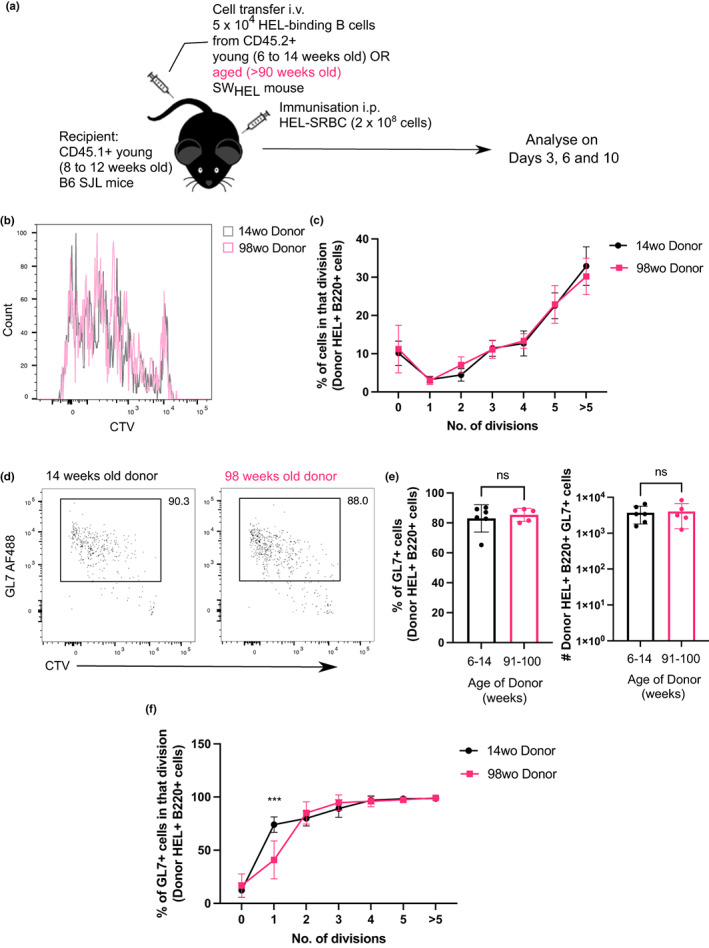
B cells from aged donor mice do not have defects in proliferation after immunisation. (a) Schematic diagram of adoptive transfer experiments to compare intrinsic function of B cells from young and aged mice in young recipient mice post‐immunisation. (b) Representative flow cytometric histograms showing the cell trace violet stains of donor HEL^+^ B220^+^ cells from either a young adult (14 weeks old) or aged (98 weeks old) mouse 3 days post‐transfer and immunisation. (c) Graph showing the percentage of donor HEL^+^ B cells in each division in recipient spleens 3 days post‐transfer and immunisation. (d) Representative flow cytometric plots for gating of GL7^+^ cells. Numbers adjacent to gates indicate percentage of donor HEL^+^ B220^+^ cells. (e) Percentage and number of GL7^+^ cells derived from donor cells from young or aged mice in recipient spleens 3 days post‐transfer and immunisation. Bar graphs show the results of one of two independent experiments (*n* = 5–6 per group/experiment). Bar height corresponds to the mean, error bars indicate standard deviation, and each symbol represents one biological replicate. Statistics were calculated using the unpaired Mann–Whitney *U* test. (f) Graph showing the percentage of GL7^+^ out of HEL^+^ Donor B cells in each division. *p*‐Value shown was generated using two‐way ANOVA with the Sidak's multiple comparisons test. Data were representative of two independent repeat experiments.

To determine if there are any cell‐intrinsic defects in B cell activation and differentiation with age *in vivo*, we employed the Switched‐HEL (SW_HEL_) adoptive transfer system. SW_HEL_ mice have two transgenes that enable them to express the high‐affinity HyHEL10 B cell receptor specific for hen egg lysozyme (HEL) from the endogenous B cell receptor locus, so the transgenic B cell receptor is capable of undergoing normal processes such as class‐switch recombination. B cells from either young adult (6–14 weeks old) or aged (>90 weeks old) SW_HEL_ mice were transferred into congenically distinct young wild‐type (WT) mice (Brink et al., 2015; Chan et al., 2012; Phan et al., 2003). These recipient mice were then immunised with HEL conjugated to sheep red blood cells (HEL–SRBC) to trigger a T‐dependent immune response (Figure 4a).This system therefore allows us to compare the ability of transferred HEL‐specific B cells from young adult or aged donor mice to undergo class‐switch recombination, enter the GC and differentiate into memory B cells or antibody‐secreting plasma cells in a young microenvironment (gating strategies shown in Figure S5A,B). First, to determine if there are any age‐dependent cell‐intrinsic defects in B cell proliferation, HEL‐binding B cells from either young or aged SW_HEL_ were labelled with cell trace violet and transferred into young recipient mice. Three days post‐immunisation, B cells from aged donor mice did not display any proliferative defects compared to those from younger mice, as shown by the similar percentages of cells in each division at this timepoint (Figure 4b,c). B cells from both young and aged donor mice also had equivalent expression of GL7 (Figure 4d,e), although early upregulation of GL7 in cells that had undergone one cell division was impaired in B cells from older donors, indicative of a delay in activation or acquisition of a GC phenotype (Figure 4f). Similarly, upon *in vitro* culture on CD40L‐expressing 40LB cells and stimulation with IL‐4 for 3 days using an induced GC culture system (Nojima et al., 2011), sorted naïve follicular B cells from aged WT mice showed no defects in their proliferation capacity (Figure S6A–C), but displayed a slight defect in acquiring the GC phenotype (CD38^−^ GL7^+^ CD95^+^) (Figure S6E). This shows that B cells from aged mice do not have gross defects in proliferation in a young microenvironment early post‐immunisation, although they may exhibit a delay in forming GC B cells.

### 2.4 B cells from aged mice have enhanced early plasma cell response and reduced GC formation early post‐vaccination

**FIGURE 5:**
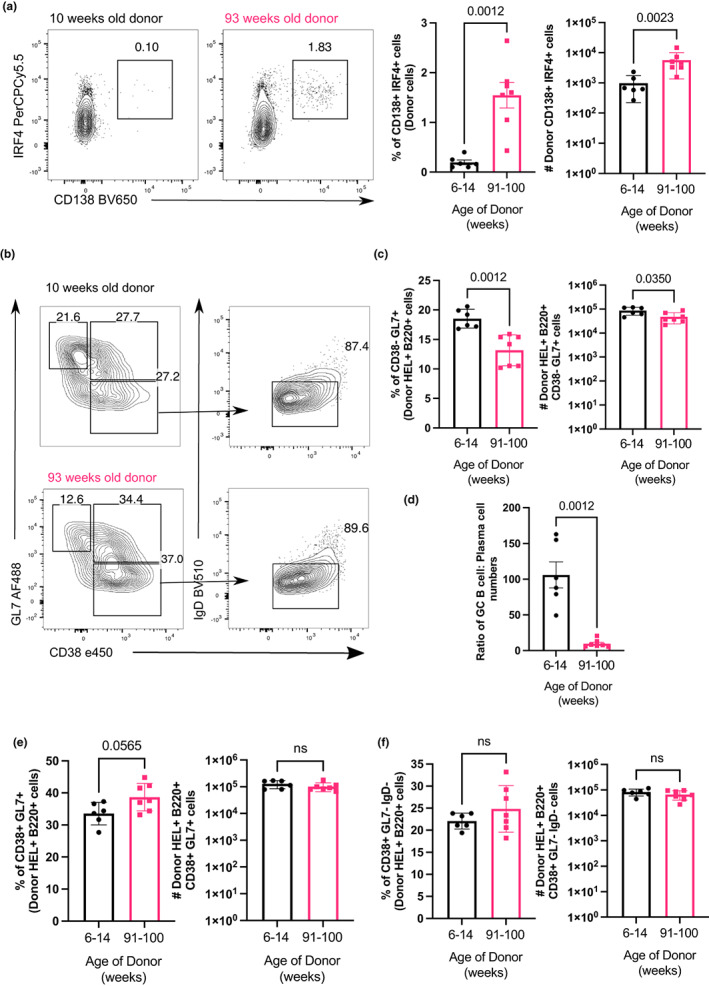
B cells from aged donor mice have enhanced extrafollicular response and reduced GC formation early post‐vaccination. (a) Representative flow cytometric plots of CD138^+^ IRF4^+^ plasma cells gated on donor cells from 10‐ or 93‐week old donor mice in recipient spleens 6 days post‐transfer and immunisation. Numbers adjacent to gates indicate percentage of donor HEL^+^ B220^+^ cells. Percentage and number of donor‐derived CD138^+^ IRF4^+^ plasma cells are plotted on the graphs on the right. (b) Representative flow cytometric plots showing gating strategies for donor‐derived GC B cells (CD38^−^ GL7^+^), activated B cells (CD38^+^ GL7^+^) and memory B cells (CD38^+^ GL7^−^ IgD^−^) from 10 weeks or 93 week old donor mice. Numbers adjacent to gates indicate percentage of donor HEL^+^ B220^+^ cells. (c) Percentage and number of HEL^+^ donor‐derived GC B cells in recipient spleen on day 6 post‐transfer and immunisation. (d) Graph showing the ratio of GC B cells: Plasma cells numbers derived from donor cells from 10 weeks or 93 weeks old donor mice. (e, f) Percentage and number of (e) CD38^+^ GL7^+^ B cells and (f) CD38^+^ GL7^−^ IgD^−^ memory B cells on day 6 post‐transfer and immunisation. Bar graphs show the results of one of two independent experiments (*n* = 5–7 per group/experiment). Bar height corresponds to the mean, error bars indicate standard deviation, and each symbol represents one biological replicate. Statistics were calculated using the unpaired Mann–Whitney *U* test.

In the first week after immunisation, some activated B cells migrate to the extrafollicular regions and differentiate into short‐lived plasma cells (Chan et al., 2009; Elsner & Shlomchik, 2020). At day 6 post‐transfer of SW_HEL_ B cells and HEL–SRBC immunization, we observed a significant increase in the percentage and number of plasma cells derived from SW_HEL_ B cells from aged donor mice compared to those from the young adult donor mice, despite no significant differences in the percentages and total numbers of donor B cells recovered in the recipient mice (Figure 5a and Figure S7A,B). In our induced GC culture system, sorted naïve follicular B cells from aged WT mice were able to differentiate into plasma cells to a similar extent as those from young mice, after culture on CD40L‐expressing 40LB cells and stimulation with IL‐4 or IL‐21 (Figure S6D,G). Together with the *in vivo* data, this suggests that B cells from aged mice do not have intrinsic defects in plasma cell differentiation after stimulation. Significantly lower percentages and numbers of GC B cells were observed among transferred SW_HEL_ B cells from aged compared to young donors (Figure 5b,c). This resulted in a significantly reduced GC B cell: plasma cell ratio among donor B cells from aged mice, relative to those from the young adult mice (Figure 5d). Together, this suggests that transferred B cells from aged mice preferentially enter the extrafollicular response at the expense of initiating the GC reaction. We observed a slight increase in percentage, but not number, of CD38^+^ GL7^+^ B cells among transferred cells from aged donors (Figure 5e). Cells with this phenotype have previously been reported to represent either precursors of memory B cells generated in the GC‐independent pathway or precursors of GC B cells (Taylor et al., 2012). As we did not observe a significant age‐related difference in the percentage of donor‐derived B cells with CD38^+^ GL7^−^ IgD^−^ memory phenotype (Figure 5f), this may suggest a delay in the entry of aged B cells into the GC response.

**FIGURE 6:**
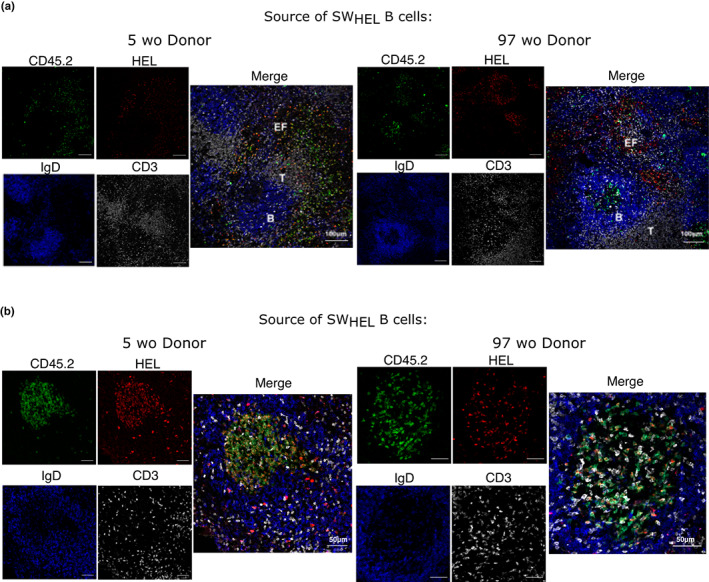
B cells from aged donor mice preferentially localize to the extrafollicular compartment early in the immune response. Confocal images of spleens of recipient mice taken 6 days post‐transfer of B cells from 5 or 97 weeks old SW_HEL_ donor mouse and HEL–SRBC immunisation. 10 μm spleen sections were stained with anti‐CD45.2 (green), anti‐HEL (red), anti‐IgD (blue) and anti‐CD3 (white) antibodies and imaged at 20× to look at the localisation of transferred cells in the (a) extrafollicular compartment and the (b) germinal centre. Data were representative of two independent repeat experiments.

Confocal imaging of the fixed spleen sections at this timepoint was also performed to compare the localization of the donor cells from young or aged mice. Corresponding to what we observed in our flow data, HEL^+^ CD45.2^+^ donor B cells from the aged mouse were more clearly observed in the extrafollicular regions of the spleen, compared with those from the young mouse (Figure 6a). Conversely, HEL^+^ donor B cells from the aged mouse in the GC appeared to be more sparse, compared to those from the young mouse (Figure 6b). Together with the flow cytometric enumeration, where we observed a lower GC B cell: plasma cell ratio derived from B cells from aged mice, this shows that B cells from aged mice preferentially enter the extrafollicular response and have a delayed early GC response, compared to B cells from young mouse.

### 2.5 B cells from aged mice have normal participation in the GC later in the response post‐vaccination

**FIGURE 7:**
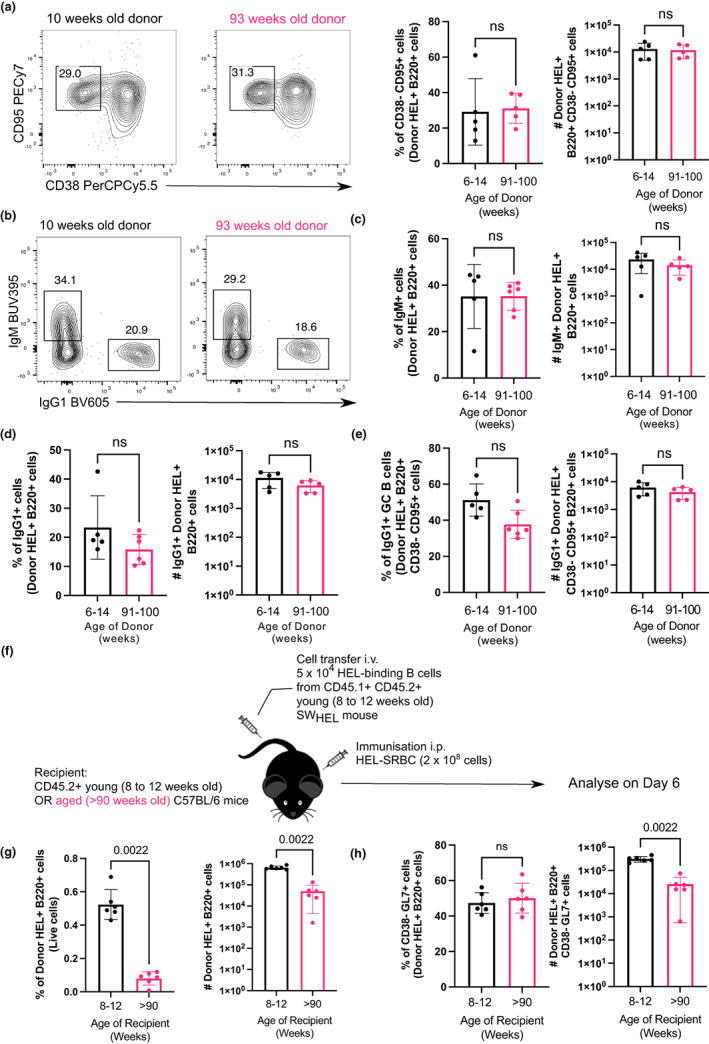
B cells from aged donor mice do not have defects in participating in the GC 10 days after immunization in young recipient mice and fewer B cells from young donor mice are recovered when transferred into aged recipient mice. (a) Representative flow cytometric plots of CD38^−^ CD95^+^ GC B cells gated on donor cells from 10‐week‐old or 93‐week‐old donor mice in recipient spleens on day 10 post‐transfer and immunisation. Numbers adjacent to gates indicate percentage of donor HEL^+^ B220^+^ cells. Percentage and number of donor‐derived GC B cells (Donor HEL^+^ B220^+^ CD38^−^ CD95^+^) are plotted on the graphs on the right. (b) Representative flow cytometric plots of IgM^+^ and IgG1^+^ donor HEL^+^ B cells. Numbers adjacent to gates indicate percentage of donor HEL^+^ B220^+^ cells. (c, d) Percentage and number of (c) IgM^+^ and (d) IgG1^+^ B cells derived from donor cells from young or aged mice in recipient spleens 10 days post‐transfer and immunisation. (e) Graph showing the percentage and number of IgG1^+^ GC B cells out of total Donor HEL^+^ B220^+^ CD38^−^ CD95^+^ cells. (f) Schematic diagram of adoptive transfer of B cells from young SW_HEL_ mice into young (8–12 weeks old) or aged (>90 weeks old) mice in which B cells response was analysed 6 days post‐transfer and immunisation. (g) Percentage and number of donor HEL^+^ B220^+^ cells in spleens of young or aged recipient mice 6 days post‐transfer and immunisation. (h) Percentage and number of donor‐derived GC B cells (Donor HEL^+^ B220^+^ CD38^−^ GL7^+^) in spleens of young or aged recipient mice 6 days post‐transfer and immunisation. Bar graphs show the results of one of two independent experiments (*n* = 5–6 per group/experiment). Bar height corresponds to the mean, error bars indicate standard deviation and each symbol represents one biological replicate. Statistics were calculated using the unpaired Mann–Whitney *U* test.

As fewer SW_HEL_ B cells from aged mice had a GC phenotype 6 days after immunisation, we assessed the impact of B cell ageing 10 days after immunisation, when the GC response has fully formed (Paus et al., 2006). There were no significant differences in the percentages and total numbers of SW_HEL_ B cells from either the young adult or aged mice recovered in the recipient mice (Figure S7C,D), nor in the frequency of GC B cells derived from the transferred cells from either the young or aged donor mouse (Figure 7a). Similarly, when naïve follicular B cells from young and aged mice were stimulated *in vitro* using the induced GC culture system for 7 days, no significant differences in cell numbers and the frequencies of GC‐like B cells were observed (Figure S6F–H). There were also no differences in the percentages and numbers of IgM^+^ and IgG1^+^ B cells, suggesting that there were no B cell‐intrinsic age‐related differences in class‐switch recombination (Figure 7b–d). Although there was a trend for a slight decrease in the percentage of IgG1^+^ GC B cells from B cells derived from the aged donor mouse, the difference was not statistically significant in either the discovery or replication experiments (Figure 7e). We also used CXCR4 and CD86 expression to distinguish dark zone (DZ) and light zone (LZ) GC B cells (Victora et al., 2010) and observed no significant differences in the DZ:LZ ratio (Figure S7E,F). This indicates that although B cells from aged mice have a delay in differentiating into GC B cells, this is not an impediment to forming GC B cells comparable to that of younger donors later in the response.

As our data have shown that aged B cells do not have intrinsic defects in proliferation or differentiation into GC B cells in a young environment, we sought to test the hypothesis that B cell‐extrinsic factors contribute to the age‐related defects in GC response by transferring SW_HEL_ B cells from a young adult mouse into young and aged WT recipient mice. Six days post‐immunisation with HEL–SRBC, B cells that were transferred into aged mice were significantly reduced in percentage recovered, consistent with a 10‐fold reduction in cell number, compared to transfers into young mice (Figure 7g). Although the small number of B cells that were present in the spleen could become GC B cells in both young and aged hosts, there was still a 10‐fold reduction in the number of GC B cells in the spleens of the aged hosts, compared to that of younger adults (Figure 7h). Together, this shows that an aged microenvironment is a key contributing factor underlying age‐related defects in the B cell response to immunisation.

## 3 DISCUSSION

Ageing is often associated with a decline in immune system function and much evidence have shown that vaccine‐induced antibody responses are diminished in older people and aged mice (Collier et al., 2021; Grubeck‐Loebenstein et al., 2009; Hill et al., 2022; Stebegg et al., 2020). An understanding of the mechanism(s) underlying this age‐related defect in vaccine response by identifying key contributing factors is vital in informing strategies to improve vaccine efficacy in older people. This study aimed to determine whether B cells from aged humans and mice have age‐associated intrinsic changes that contribute to age‐related defects in GC response and plasma cell formation upon vaccination. Here, we show that plasma cell differentiation is intact in B cells from older people *in vitro*, and in B cells from aged mice *in vivo* and *in vitro* after T‐dependent stimulation. This is consistent with previous studies in mice, which showed that transgenic B cells from aged mice transferred into young recipient mice were able to differentiate into high‐affinity, isotype‐switched plasma cells in the spleen after immunisation *in vivo*, comparable to those from young mice (Dailey et al., 2001).

While we did not observe any age‐related cell‐intrinsic defects in human B cell proliferation and differentiation into plasma cells, we did observe a decrease in the percentage of B cells in the peripheral blood of older donors compared to younger donors, as previously reported (Frasca et al., 2008; Frasca & Blomberg, 2009). This may suggest that changes in B cell number, rather than intrinsic defects in their function, contribute to attenuated antibody responses in older people. In both our study and a larger, independent cohort, we did not observe any significant differences in the proportions of naïve and memory B cells in the peripheral blood. Other studies have reported an age‐related increase in the percentage of naïve B cells and decrease in percentage of CD27^+^ memory B cells in the peripheral blood of older humans (Breitbart et al., 2002; Chong et al., 2005; Frasca et al., 2011; Shi et al., 2005). One possible explanation for this discrepancy is that the older donors (>65 years old) in our cohorts are healthy community dwelling people, who were able to go to the blood donation centre to donate blood, and therefore they might not have age‐dependent differences in B cells proportions reported in other studies that are associated with frailty (Breitbart et al., 2002). Furthermore, there are also contradicting observations in existing literature; while Shi et al. (2005) reported a significant age‐related decrease in the percentage of IgD^+^ CD27^+^ cells, Frasca et al. (2011) reported no age‐related differences in the percentage of IgM memory B cells, similar to what we observed. This suggests that observations of age‐related changes in cell proportions are highly dependent on cohort and experimental set‐up and subject to high variability.

We also observed that naïve and memory B cells from older people have lower basal expression levels of IRF4 than those from young donors. IRF4 is a transcription factor lowly expressed in naïve cells, absent in GC B cells and upregulated in plasma cells (Willis et al., 2014). In particular, IRF4 has been shown to be important for regulating the proliferative capacity of B cells during activation and is required for early GC B cells formation in a B cell‐intrinsic manner (Patterson et al., 2021; Willis et al., 2014). Thus, the lower expression of IRF4 by B cells from older donors may result in early proliferative defects, as previously reported (Blaeser et al., 2008) and contribute to the reduced early GC response, as we observed in our mouse studies. Nevertheless, our *in vitro* differentiation assay data shows that B cells from older donors do not have a defect in proliferating and differentiating into plasma cells after 6 days stimulation with CD40L and IL‐21, suggesting that the reduction in IRF4 does not have a significant impact on B cell response to T‐dependent stimulation. While naïve and memory B cells from older humans did not display any defects in upregulating costimulatory molecules (CD80, CD86 and HLA‐DR) and activation markers (CD69 and IL‐21R) after stimulation with CD40L and IL‐21, naïve B cells from older humans had a tendency to express higher levels of HLA‐DR. This is consistent with a study that shows higher expression of HLA‐DR in PBMCs of older people (Wu et al., 2011), although the cause and effect of the increased upregulation of HLA‐DR on B cells in ageing are not known.

The SW_HEL_ adoptive transfer system allowed us to compare the function of B cells from young or aged donor mouse in responding to vaccination *in vivo* in a young environment. In most of the transfer experiments we performed, we did not observe any reproducible differences in the percentage and numbers of HEL‐binding donor B cells we could recover in the spleens of the recipient mice. This suggests that B cells from aged mice are unlikely to have defects in their survival in the recipient mice and in homing to the spleen. This is consistent with previous studies, which showed that transferred B cells from young and aged mice have no differences in migration to draining lymph nodes and spleens after viral infection (Richner et al., 2015). Similar to our human *in vitro* data, we also show that B cells from aged mice do not have defects in proliferating early post‐vaccination *in vivo*. Naïve follicular B cells from aged mice also had similar proliferative capacity as those from young mice when cultured in the induced GC system for 3 days on 40LB cells with IL‐4. Previous studies have, however, suggested that transferred B cells from aged donors have reduced expansion *in vivo* following immunisation, though the difference was not statistically significant (Dailey et al., 2001). B cells from aged mice have also been shown to have a reduced proliferative capacity compared to those from young mice, when stimulated *in vitro* with anti‐CD40 and IL‐4 or LPS (Blaeser et al., 2008). This may suggest that B cells can have different rates of proliferation in response to certain antigens or stimulants, or that certain B cell subsets other than those of the naïve follicular phenotype have age‐related defects in proliferation.

Early after immunisation, we observed that transferred B cells from aged mice were more likely to have an extrafollicular plasma cell phenotype than a GC B cell phenotype. Immunofluorescence staining of the transferred B cells shows that B cells from aged donor mice were more easily observed in the extrafollicular compartment than in the GC, compared to those from the young adult mice. Previous studies have shown that follicular B cells from aged mice have defects in migrating towards CXCL13, compared to young B cells, in *in vitro* transwell assays (Turner & Mabbott, 2017). As such, the reduced early GC formation that we observe among B cells from aged mice could be due to their intrinsic defects in localizing to the B cells follicle to initiate and enter the GC reaction. Nevertheless, at the peak of the GC response 10 days post‐immunisation, B cells from aged mice showed no detectable defects in GC responses and class‐switch recombination, similar to what has been previously reported (Dailey et al., 2001; Yang et al., 1996). This shows that cell‐intrinsic changes in B cells with age may delay the GC response, but are unlikely to contribute to poor GC response at its peak. These observations were also recapitulated when sorted naïve follicular B cells from young and aged WT mice were stimulated *in vitro* using the induced GC culture, where naïve B cells from aged donor had an early defect in adopting the GC‐like phenotype at day 3 post‐stimulation but this defect was no longer observed at day 7 post‐stimulation.

Our observations that there are no significant age‐related defects in class‐switch recombination are concordant with previous *in vivo* studies by (Dailey et al., 2001) and *ex vivo* studies on aged mouse follicular B cells by (Russell Knode et al., 2019), but are not consistent with other studies that reported reduced class‐switch recombination in aged splenic mouse B cells when stimulated *in vitro* with CD40L and IL‐4 (Frasca et al., 2004, 2007). This may suggest some differences in B cells response *in vitro* and *in vivo*, and/or that there are some factors in the young microenvironment in vivo that can compensate for the reduced expression of AID in aged B cells linked with impaired class switch recombination. For example, it has been previously suggested that inflammatory cytokines like TNFα can interfere with B cells response, specifically in downregulating the E47 transcription factor and AID enzyme, which are required for class‐switch recombination (Frasca et al., 2012). As such, the lower levels of inflammatory cytokines in young recipient mice could account for the lack of defects in class‐switch recombination by aged B cells in our adoptive transfer experiments. It might also be possible that only specific subsets of B cells have intrinsic age‐related defects in class switching *in vitro* (Russell Knode et al., 2019).

When transgenic B cells from a young SW_HEL_ mouse were transferred into young and aged recipient mice, very few of the transferred HEL^+^ B cells were detectable in the aged recipient mice, compared to those transferred into a young environment. These observations match those from heterochronic parabiosis studies, where the circulatory systems of young and aged mice are surgically conjoined; B cells from young mice displayed a significant defect in mounting a GC response in an aged lymph node compared to those in a young environment (Denton et al., 2022). This, together with data from our adoptive transfer experiment, suggests that the age of the lymphoid environment, rather than the age of B cells, contributes more to defects in GC formation during ageing.

The data we have presented here collectively show that B cells from aged humans and mice do not have intrinsic defects in proliferation, activation and differentiation into plasma cells after T‐dependent stimulation. In addition, aged B cells do not have defects in undergoing class‐switch recombination and mounting a GC reaction in young recipient mice. Conversely, fewer antigen‐specific young B cells were recovered when transferred into an aged environment. This suggests that B cell‐extrinsic factors like poor T cell help and the aged microenvironment are likely more dominant contributors to the age‐related defects in GC response and reduction in antibody response in older individuals (Eaton et al., 2004; Lefebvre et al., 2012; Stebegg et al., 2020; Yang et al., 1996). Some factors that have been implicated in causing reduced GC responses in aged mice include defects in the priming of T cells by dendritic cells, impaired T cells help provided to B cells and intrinsic defects in stromal cell activation and function during ageing (Denton et al., 2022; Eaton et al., 2004; Stebegg et al., 2020).

## 4 EXPERIMENTAL PROCEDURES

### 4.1 Human PBMC preparation and B cell isolation

Leukapheresis samples (NC24) from healthy donors were obtained from either the National Health Service Blood and Transplant Service following ethical approval (approved by the UK Health Research Authority REC reference 19/NE/0060) or the NIHR BioResource UK local research ethics committee approval (REC reference 14/SC/1077), using the facilities of the Cambridge Bioresource (REC reference 04/Q0108/44). Younger donors were classified as individuals who were 20 to 34 years old while older donors were >64 years old. Peripheral blood mononuclear cells (PBMCs) were isolated by density gradient centrifugation using Histopaque‐1077 (Sigma), before being cryopreserved in 90% foetal calf serum (FCS) with 10% DMSO and stored in liquid nitrogen until required. Cryopreserved samples were thawed and rested in medium (RPMI‐1640 with 10% FCS, 100 U/ml penicillin, 100 μg/ml streptomycin and 0.1 mg/ml DNAse) for 1 h at 37°C. Cells were then counted and stained with Cell‐Trace Violet (Invitrogen) at a final concentration of 5 μM/ml per 1 × 10^7^ cells in PBS for 20 min at 37°C. After incubation, the reaction was quenched by adding five times the original staining volume of culture medium (RPMI‐1640 with 10% FCS) to the cells. B cells were then purified and enriched from the PBMCs by negative selection using a MagniSort™ human B cell enrichment kit (Invitrogen) following manufacturer's protocol. Cells were then resuspended at 4 × 10^6^ cells/ml in PBS with 2% FCS and 2 mM EDTA for Fluorescence‐Activated Cell Sorting.

### 4.2 Purification of naïve and memory B cells by fluorescence activated cell sorting

B cells were incubated with an antibody cocktail containing L/D marker (eBioscience Fixable Viability Dye eFluor 780) and antibodies CD10 BUV737 (BD #612826), CD20 PECy7 (BioLegend #302312) and CD27 BV711 (BioLegend #302834) and IgD BUV395 (BD #563813) for 30 min at 4°C. Labelled B cells were sorted into naïve (CD10^−^CD20^+^CD27^−^IgD^+^) and memory (CD10^−^CD20^+^CD27^+^ and CD10^−^CD20^+^CD27^−^IgD^−^) B cells using the FACSAria™ Fusion sorter (BD Biosciences) (gating strategy shown in Figure S1A).

### 4.3 In vitro human plasma cell differentiation assay

Sorted naïve and memory B cells were cultured in a round‐bottom 96‐well plate (Corning) at a density of 2.5 × 10^4^ cells/200 μl in complete medium (RPMI‐1640 with 10% FCS, 100 U/ml penicillin and 100 μg/ml streptomycin, GlutaMAX [Thermo Fisher Scientific], sodium pyruvate [Sigma], HEPES [Sigma], non‐essential amino acids [Sigma], 2‐mercaptoethanol [Sigma], amphotericin B [Thermo Fisher Scientific] and apotransferrin [T1428, Sigma]). Stimulated B cells were incubated with 200 ng/ml CD40L (R&D Systems, 6245‐CL) and 50 ng/ml IL‐21 (Peprotech, 200‐21‐10), or with 25 ng/ml IL‐2 (Peprotech, 200‐02019), or 2.5 μg/ml CpG ODN2006 (Invivogen), in 200 μl complete medium for the specified number of days. For cultures beyond 6 days, 100 μl of old media was removed and replaced with fresh media with the same concentrations of stimulants every 4 days. The HA‐tagged CD40L was incubated with 50 ng/ml anti‐HA antibody (Abcam, ab1421) for 15 min at 37°C prior to stimulation of cells to cross‐link the proteins. The cells were incubated in 37°C incubator with 5% CO_2_.

### 4.4 Flow cytometric analysis of human cell cultures

Cells were resuspended and transferred to a v‐bottom 96‐well plate (Corning). Cells were washed once with PBS with 2% FCS and 2 mM EDTA, before being incubated with anti‐human CD32 FcR block (BioLegend) for 10 min at 4°C. Surface antibody staining was performed for 30 min at 4°C in Brilliant Stain Buffer (BD Biosciences #563794). After incubation, cells were washed and fixed using eBioscience Foxp3‐staining fixation/permeabilization buffer for 20 min at 4°C. Cells were then stained with PE‐conjugated anti‐IRF4 antibody in 1× eBioscience permeabilization buffer for 1 h at 4°C. Samples were washed with PBS with 2% FCS before they were acquired on a LSRFortessa (BD Biosciences). Flow cytometry data were analysed using FlowJo v10 software (Tree Star). The average number of divisions undergone by proliferating cells or proliferation index was computed using the ‘Proliferation’ tool on Flowjo v10 (Tree Star). For determination of number of plasma cells, 25 μl of ACBP‐50‐10 particles (Spherotech AccuCount Blank Particles Lot AJ01, 51,200 per 50 μl) were added to each tube before acquisition. The number of plasma cells in each sample was then calculated using this formula: (*A*/*B*) × *C*, where *A* = number of events for test samples, *B* = number of events for the ACBP‐50‐10 particles, *C* = number of ACBP‐50‐10 particles per 25 μL for this lot (25,600). The antibodies used are listed in Table 1. The Live/Dead stain, anti‐CD14, anti‐CD16 and anti‐CD3 antibodies were included in the APC‐e780 channel as the DUMP channel.

**TABLE 1:** List of antibodies used for flow cytometric analysis of human cells

		Company and clone	Identifier	Dilution
Panel for human plasma cell differentiation				
1	APC‐e780‐coupled Live/Dead	eBioscience	65‐0865‐14	1:10000
2	APC‐e780‐coupled anti‐human CD14	eBioscience (61D3)	470149‐42	1:400
3	APC‐e780‐coupled anti‐human CD16	eBioscience (eBioCB16)	47‐0168‐42	1:400
4	APC‐e780‐coupled anti‐human CD3	eBioscience (UCHT1)	47‐0038‐42	1:400
5	BB515‐coupled anti‐human CD19	BD (HIB19)	564456	1:200
6	APC‐coupled anti‐human CD38	eBioscience (HIT2)	17‐0389‐42	1:400
7	BV711‐coupled anti‐human CD27	BioLegend (0323)	302834	1:400
8	PeCy7‐coupled anti‐human CD20	BioLegend (2H7)	302312	1:400
9	BUV737‐coupled anti‐human IgD	BD (IA6‐2)	563813	1:100
10	PE‐coupled IRF4	BioLegend (IRF4.3E4)	646404	1:200
Panel for costimulatory molecules expression				
1	APC‐e780‐coupled Live/Dead	eBioscience #65‐0865‐14	65‐0865‐14	1:10000
2	APC‐e780‐coupled anti‐human CD14	eBioscience (61D3)	470149‐42	1:400
3	APC‐e780‐coupled anti‐human CD16	eBioscience (eBioCB16)	47‐0168‐42	1:400
4	APC‐e780‐coupled anti‐human CD3	eBioscience (UCHT1)	47‐0038‐42	1:400
5	BB515‐coupled anti‐human CD19	BD (HIB19)	564456	1:200
6	BV711‐coupled anti‐human CD27	BioLegend (0323)	302834	1:400
7	PeCy7‐coupled anti‐human CD20	BioLegend (2H7)	302312	1:400
8	BV510‐coupled anti‐human IgD	BD (IA6‐2)	563813	1:200
9	PerCPe710‐coupled anti‐human IL‐21R	eBioscience (2SX21R)	46‐3601‐4	1:200
10	PE‐coupled anti‐human CD80	eBioscience (2D10.4)	12‐0809‐42	1:400
11	PECf594‐coupled anti‐human CD86	BD (2331)	562390	1:400
12	BUV395‐coupled anti‐human HLA‐DR	BD (G46‐6)	565972	1:400
13	BV786‐coupled anti‐human CD69	BD (FN50)	310932	1:200
Panel for phenotypic analysis				
1	APC‐e780‐coupled Live/Dead	eBioscience	65‐0865‐14	1:10000
2	APC‐e780‐coupled anti‐human CD14	eBioscience (61D3)	470149‐42	1:400
3	APC‐e780‐coupled anti‐human CD16	eBioscience (eBioCB16)	47‐0168‐42	1:400
4	APC‐e780‐coupled anti‐human CD3	eBioscience (UCHT1)	47‐0038‐42	1:400
5	BB515‐coupled anti‐human CD19	BD (HIB19)	564456	1:200
6	APC‐coupled anti‐human CD38	eBioscience (HIT2)	17‐0389‐42	1:400
7	BV711‐coupled anti‐human CD27	BioLegend (0323)	302834	1:400
8	PeCy7‐coupled anti‐human CD20	BioLegend (2H7)	302312	1:400
9	BUV3955‐coupled anti‐human IgD	BD (IA6‐2)	563813	1:100
10	PerCPe710‐coupled anti‐human IL‐21R	eBioscience (2SX21R)	46‐3601‐4	1:200
11	PE‐coupled IRF4	BioLegend (IRF4.3E4)	646404	1:200
12	BV450‐coupled CD40	BD (5C3)	BD 561219	1:400
Surface stain for spectral flow cytometric analysis				
1	BUV563‐coupled anti‐human FcRL5	BD (509F6)	749598	1:500
2	BUV615‐coupled anti‐human CD19	BD (HIB19)	751273	1:2000
3	BV480‐coupled anti‐human CD21	BD (B‐Ly4)	746613	1:2000
4	SparkNIR686‐coupled anti‐human CD20	BioLegend (2H7)	302366	1:2000
5	ViaKrome808‐coupled Live/Dead	Beckman Coulter	C36628	1:2000
6	APC‐Fire750‐coupled anti‐human IgD	BioLegend (IA6‐2)	348328	1:2000
Intracellular stain for spectral flow cytometric analysis				
7	BUV395‐coupled anti‐human CD27	BD (L128)	563816	1:2000
8	BUV737‐coupled anti‐human CD10	BD (HI10a)	741825	1:1000
9	AF532‐coupled anti‐human IgM	NOVUS (IM373)	NBP2‐34650AF532	1:1000
10	SparkBlue574‐coupled anti‐human CD3	BioLegend (SK7)	344851	1:1000

### 4.5 Phenotypic analysis of human PBMCs

Cryopreserved samples from younger donors (18–34 years old) or older donors (68–76 years old) were thawed and rested as described earlier. Cells were washed twice with PBS with 2% FCS and 2 mM EDTA, before being incubated with anti‐human CD32 FcR block (BioLegend) for 10 min at 4°C. Surface antibody staining was performed for 30 min at 4°C in Brilliant Stain Buffer (BD Biosciences #563794). The antibodies used for this analysis are listed in Table 1. The Live/Dead marker, anti‐CD14, anti‐CD16 and anti‐CD3 antibodies were included in the APC‐e780 channel as the dump channel. Cells were washed and fixed using eBioscience Foxp3 staining fixation/permeabilization buffer for 20 min at 4°C. Cells were then stained with PE‐conjugated anti‐IRF4 antibody in 1× eBioscience permeabilization buffer for 1 h at 4°C. Samples were acquired on a LSRFortessa (BD Biosciences) and flow cytometry data were analysed using FlowJo v10 software (Tree Star).

A second, independent cohort of younger (18–36 years old) and older donors (66–98 years old) was analysed using spectral flow cytometry. Cryopreserved cells were thawed as above. Surface staining was performed for 2 h at 4°C in Brilliant Stain Buffer (BD Biosciences #563794), in the presence of anti‐human CD32 FcR block (BioLegend). Cells were then washed and fixed using eBioscience Foxp3‐staining fixation/permeabilization buffer for 20 min at 4°C, before being stained with intracellular antibodies in 1 × eBioscience permeabilization buffer overnight at 4°C. Stained cells were acquired on a Cytek™ Aurora. Cells for single colour controls were prepared in the same manner as the fully stained samples. Manual gating of flow cytometry data was done using FlowJo v10.8 software (Tree Star). The antibodies used for both surface and intracellular staining are listed below (Table 1).

### 4.6 Enzyme‐linked immunosorbent assay (ELISA) for Human IgG, IgM and IgA

The ELISA plates (Thermo Fisher Scientific 96F Maxisorp #456537) were coated with 0.5 μg/ml anti‐human IgG (BioLegend #410701) or 0.25 μg/ml anti‐human IgM (Southern BioTech #2020–01) or 0.167 μg/ml anti‐human IgA (Southern BioTech #2050‐01) diluted in PBS for 2 h at room temperature. After incubation, plates were washed three times with wash buffer containing 0.05% Tween 20 in PBS and blocked with 2% BSA in PBS overnight at 4°C. The next day, plates were washed three times in wash buffer (PBS containing 0.05% Tween 20). Samples and standards diluted in RPMI + 10% FCS were added and plates were incubated for 2 h at room temperature. After washing the plates three times with wash buffer, 50 μl of anti‐human biotinylated IgG (BioLegend #409307, 1:1500 in PBS) or anti‐human biotinylated IgM (Southern BioTech #2020‐08, 1:1500 in PBS) or anti‐human biotinylated IgA (Southern BioTech #2050‐08, 1:3000 in PBS) were added for 2 h at room temperature. Plates were washed three times with wash buffer, before being incubated with 50 μl of Streptavidin‐HRP (GE Healthcare #RPN1231V) for 30 min at room temperature. The plates were developed with 100 μl/well TMB (Biolegend #421101) for up to 20 min, and the reaction was stopped with 50 μl/well 2.5 M H2SO4. PHERAstar FS microplate reader (BMG Labtech) was used to measure absorption at 450 and 570 nm. Absorbance values at 570 nm were subtracted from absorbance values at 450 nm. Absorbance values from diluted samples were calculated and the antibody concentration was calculated by interpolation from a standard curve generated on the same plate.

### 4.7 Animals

SW_HEL_ and WT C57BL/6 mice were bred and maintained in the Babraham Institute Biological Support Unit (BSU), where SW_HEL_ mice were also aged. No primary pathogens or additional agents listed in the FELASA recommendations (Mahler et al., 2014) were detected during health‐monitoring surveys of the stock holding rooms. Ambient temperature was ~19–21°C and relative humidity 52%. Lighting was provided on a 12‐h light: 12‐h dark cycle including 15 min ‘dawn’ and ‘dusk’ periods of subdued lighting. After weaning, mice were transferred to individually ventilated cages with 1–5 mice per cage. Mice were fed CRM (P) VP diet (Special Diet Services) ad libitum and received seeds (e.g., sunflower, millet) at the time of cage cleaning as part of their environmental enrichment. All the mouse experimentation was approved by the Babraham Institute Animal Welfare and Ethical Review Body. Animal husbandry and experimentation complied with existing European Union and United Kingdom Home Office legislation and local standards (PPL: P4D4AF812). Young adult SW_HEL_ mice were 5–14 weeks old, and aged SW_HEL_ mice were at least 90 weeks old when used for experiments. Young recipient B6.SJL or C57BL/6 mice were 8–12 weeks old at the time of immunisation.

### 4.8 Conjugation of HEL to SRBC

Fresh sheep red blood cells (SRBC) in Alsever's solution (TCS Biosciences) were used in each experiment. 5 ml of SRBC were sterile transferred into a 50 ml Falcon tube and washed 4 times with 1× PBS. The cells were then washed once in 20 ml conjugation buffer (0.35 M mannitol and 0.01 M NaCl) before being resuspended in 4 ml of conjugation buffer. 0.5 ml of hen egg lysozyme (HEL, Sigma‐Aldrich) was then added to the cells to a final concentration of 0.2 mg/ml and the cells were placed on ice with rocking for 10 min. 0.5 ml of 100 mg/ml of N‐(3‐Dimethylaminopropyl)‐N′‐ethylcarbodiimide hydrochloride (EDCI, Sigma Aldrich E‐7750) was added to the cells and the cells were further incubated on ice with rocking for 30 min. The cells were subsequently washed 4 times with PBS, before being diluted to a concentration of 2 × 10^9^ cells/ml. All spins between washes were performed at 1111 g for 5 min at 4°C, with brakes turned off. Successful conjugation of HEL to SRBC was confirmed by flow cytometric analysis using Alexa Fluor® 647‐conjugated HyHEL9 antibody at 1:1000 dilution. Mock‐conjugated SRBC in which HEL was omitted from the conjugation reaction was used as a negative control.

### 4.9 SW_HEL_ adoptive transfers

Single cell suspensions of spleen and mesenteric and peripheral lymph nodes from a young 6–14 week old adult and an >90 week old aged SW_HEL_ mice were obtained by pressing the tissues through a 70 μm mesh in PBS with 2% foetal bovine serum. Cell numbers and viability were determined using a CASY TT Cell Counter (Roche). For analysis of proliferative capacity of B cells, the splenocytes were stained with CellTrace™ Violet (Invitrogen C34557) according to manufacturer's instructions prior to transfer. The percentage of HEL‐binding B cells in each cell suspension was determined by flow cytometric analysis using Alexa Fluor® 647‐conjugated HEL and BV785‐conjugated anti‐mouse B220 antibodies. The cell suspension were then diluted in appropriate volumes of PBS to obtain a final concentration of 5 × 10^5^ HEL‐binding B cells/ml. 100 μl of 5 × 10^4^ HEL‐binding B cells from young and aged donor SW_HEL_ mice were then injected intravenously into their respective WT recipients. Recipient mice were also immunised intraperitoneally with 100 μl of 2 × 10^8^ HEL‐conjugated SRBC. The spleens of recipient mice were harvested on the indicated days post‐immunisation and flow cytometry was performed.

### 4.10 Flow cytometric analysis of mouse splenocytes

Single cell suspensions of half of the dissected spleens from the recipient mice were obtained by pressing the tissues through a 70 μm mesh in PBS with 2% FCS. Cell numbers and viability were determined using a CASY TT Cell Counter (Roche). 15–20 × 10^6^ cells were stained for each panel. Cells were first incubated with FcR block (anti‐mouse CD16/32, clone 93, eBioscience) for 10 min at 4°C. Surface antibody staining was then performed for 60 min at 4°C in Brilliant Stain Buffer (BD Biosciences #563794). For Panel 1 consisting of anti‐CD138 biotinylated antibody, the cells were then stained with BV650‐conjugated streptavidin for 30 min at 4°C. For intranuclear staining, cells were fixed first with BD Cytofix/Cytoperm™ Fixation/Permeabilization solution (BD Biosciences 554714) for at least 20 min before being stained with the anti‐IRF4, anti‐Ki67 and anti‐Casp3 antibody mix for 1 h at 4°C in 1× BD Cytofix/Cytoperm™ Perm/Wash buffer. Samples were acquired on a LSRFortessa (BD Biosciences) and flow cytometry data were analysed using FlowJo v10 software (Tree Star). The average number of divisions undergone by proliferating cells or proliferation index was computed using the ‘Proliferation’ tool on Flowjo v10 (Tree Star). The antibodies used for the two panels are shown in Table 2.

**TABLE 2:** List of antibodies used for flow cytometric analysis of mouse cells

		Company and clone	Identifier	Dilution
Antibodies in panel 1				
1	APC‐e780‐coupled Live/Dead	eBioscience	65‐0865‐14	1:10000
2	BV785‐coupled anti‐mouse B220	BioLegend (RA3‐6B2)	103246	1:300
3	PeCy7‐coupled anti‐mouse CD45.1	Invitrogen (A20)	25‐0453‐82	1:200
4	BV605‐coupled anti‐mouse CD45.2	BioLegend (104)	109841	1:200
5	E450‐coupled anti‐mouse CD38	Invitrogen (90)	48‐0381‐82	1:200
6	A647‐coupled HEL	Conjugated in‐house	NA	1:2000
7	BUV395‐coupled anti‐mouse CD19	BD (1D3)	565965	1:100
8	Biotin‐coupled anti‐mouse CD138	BioLegend (281‐2)	142512	1:100
9	BV650‐coupled streptavidin	BioLegend	405231	1:200
10	BV510‐coupled anti‐mouse IgD	BD (11‐26c.2a)	563110	1:200
11	AF488‐coupled GL‐7	Invitrogen (GL‐7)	53‐5902‐82	1:100
12	PerCPCy5.5‐coupled IRF4	BioLegend (IRF4.3E4)	646416	1:50
13	AF700‐coupled anti‐mouse Ki67	BioLegend (16A8)	652420	1:100
Antibodies in panel 2				
1	L/D Fixable Blue Dead Cell Stain	Invitrogen	L34961	1:1000
2	BUV737‐coupled anti‐mouse CD4	BD (RM4‐5)	612844	1:200
3	BV650‐coupled anti‐mouse B220	BioLegend (RA3‐6B2)	103241	1:200
4	BV785‐coupled anti‐mouse CXCR5	BioLegend (L138D7)	145523	1:200
5	APCe780‐coupled anti‐mouse PD1	Invitrogen (J43)	47‐9985‐82	1:200
6	AF700‐coupled anti‐mouse CD45.2	Invitrogen (104)	56‐0454‐82	1:200
7	PE‐ef610‐coupled anti‐mouse CD45.1	Invitrogen (A20)	61‐0453‐82	1:200
8	PerCPCy5.5‐coupled anti‐mouse CD38	BioLegend (90)	102722	1:200
9	PeCy7‐coupled anti‐mouse CD95	BD (Jo2)	557653	1:200
10	A647‐coupled HEL	Conjugated in‐house	NA	1:2000
11	BUV395‐coupled anti‐mouse IgM	BD (R6‐60.2)	564025	1:100
12	BV510‐coupled anti‐mouse CD86	BD (GL1)	563077	1:50
13	PE‐coupled anti‐mouse CXCR4	BioLegend (L276F12)	146506	1:100
14	E450‐coupled anti‐mouse IgD	Invitrogen (11‐26c)	48‐5993‐82	1:100
15	BV605‐coupled anti‐mouse IgG1	BD (A85‐1)	563285	1:100

### 4.11 Confocal imaging

Half of the spleen harvested 6 days post‐transfer and immunisation was fixed in periodate‐lysine‐paraformaldehyde (PLP) containing 1% (v/v) PFA (Sigma #P6148), 0.075 M L‐Lysine (Sigma #L5501), 0.37 M Na_3_PO_4_ (pH 7.4) (Sigma #342483) and 0.01 M NaIO_4_ (Sigma #210048), for 4 h at 4°C. After fixation, the samples were dehydrated in 30% sucrose (Sigma #S0389) overnight, before being embedded in optimum cutting temperature medium (VWR #25608‐930) on dry ice and stored at −80°C. The frozen spleen samples were cut into 10 μm sections using a cryostat (Leica Biosystems) at −20°C and again stored at −80°C. For staining, the slides were first air‐dried and then hydrated in 0.5% Tween 20 in PBS (PBS‐T) for 10 min at 20–22°C. Slides were then permeabilised with 2% Triton (Sigma #X100) diluted in PBS for 10 min. After washing three times with PBS‐T, slides were blocked in blocking buffer (PBS containing 2% BSA and 10% normal goat serum) for 30 min. After washing three times with PBS‐T, the slides were blocked with 40 μg/ml unconjugated Fab fragment goat anti‐mouse IgG (H + L) (#115‐007‐003, Jackson ImmunoResearch) for 30 min at room temperature. After blocking, the slides were first stained with biotin anti‐mouse CD45.2 (clone 104, BioLegend, 1:200) for 30 min at room temperatures. The slides were washed once with PBS‐T for 10 min before being incubated with BV421‐conjugated Streptavidin (BioLegend, 1:1000) for 20 min at room temperature. After washing once with PBS‐T for 10 min, slides were stained with BV510‐conjugated rat anti‐mouse IgD (clone 11‐26c.2a, BD; 1:100), AF647‐conjugated HEL (Made in‐house, 1:5000) and purified hamster anti‐mouse CD3ε (clone 500A2, BioLegend, 1:100) for 60 min at room temperature. After washing once again with PBS‐T for 10 min, the slides were incubated with AF568‐conjugated goat anti‐hamster IgG (#A‐21112, Life Technologies; 1:1000) for 30 min at room temperature. The slides were washed twice with PBS‐T and once with PBS before being mounted using Hydromount‐mounting medium (National Diagnostics #HS‐106). Slides were dried overnight for the mounting medium to set. Images were acquired using a Zeiss 780 confocal microscope using 10× and 20× objectives. Image analysis was performed using ImageJ. This protocol was adapted from an optimized protocol for staining and imaging of GCs in secondary lymphoid tissues of vaccinated mice (Fra‐Bido et al., 2021).

### 4.12 Induced germinal centre culture

Naïve follicular B cells (Live B220^+^ IgD^+^ CD93^−^ CD23^+^) were first sorted from spleens of young (8–12 weeks old) and aged (>90 weeks old) mice using fluorescence‐activated cell sorting. For analysis of proliferative capacity of B cells, the splenocytes were stained with CellTrace™ Violet (Invitrogen C34557) according to manufacturer's instructions. B cells were then incubated with an antibody cocktail containing L/D marker (eBioscience Fixable Viability Dye eFluor 780) and antibodies IgD AF647 (BioLegend #405708), CD93 PE (BioLegend #136504) and CD23 FITC (BioLegend #101605) for 30 min at 4°C. Purified naïve follicular B cells were cultured in a 12‐well plate at a density of 4 × 10^4^ cells/well in the presence of 40LB cells (1 × 10^5^ cells/well) that had been irradiated with 120 Gy γ‐ray in 4 ml RPMI‐1640 medium (Sigma) supplemented with 10% FCS, 5.5 × 10^−5^ M 2‐ME, 10 mM HEPES, 1 mM sodium pyruvate, 100 units/ml penicillin, and 100 μg/ml streptomycin (GIBCO). rIL‐4 (2 ng/ml; Peprotech) was added to the primary culture for 3 or 4 days for the 3‐day or 7‐days culture, respectively. On day 4, the cells were re‐seeded into 24‐well plates with a new feeder layer and cultured with rIL‐4 (2 ng/ml; Peprotech) or rIL‐21 (5 ng/ml; Peprotech) for another 3 days. For the 7‐days culture, media was refreshed on days 3 and 6 post‐stimulation. The cells were incubated in 37°C incubator with 5% CO_2_. Cells were counted and analysed using flow cytometric analysis after 7 days of stimulation.

### 4.13 Statistical analysis

All the experiments were performed twice or more (3–5 human donors per group or 5–7 recipient mice per group). Statistical analysis was performed using Prism v9 software (GraphPad). Differences between experimental groups were assessed using the unpaired Mann–Whitney *U* test or two‐way ANOVA with Sidak's multiple comparisons test. The *p* values were considered significant when <0.05.

## AUTHOR CONTRIBUTIONS

Conceptualization, M.A.L. and J.L.L.; Methodology, J.L.L., S.F., A.R.B., S.I., D.L.H. and M.A.L.; Investigation, J.L.L., S.F., A.R.B., S.I. and M.A.L.; Writing – Original draft preparation, J.L.L. and M.A.L.; Writing – Review and Editing, all authors; Funding Acquisition, M.A.L.

## CONFLICT OF INTEREST

The authors declare that they have no conflicts of interest.

## Supporting information


Figure S1
Figure S2
Figure S3
Figure S4
Figure S5
Figure S6
Figure S7

## Data Availability

Data sharing is not applicable to this article as no new data were created or analyzed in this study.
