# Supplementary material for: B cell‐intrinsic changes with age do not impact antibody‐secreting cell formation but delay B cell participation in the germinal centre reaction
Source: Aging Cell. 2022 Aug 18;21(9):e13692. doi: 10.1111/acel.13692 (PMC9470890; doi:10.1111/acel.13692)
Supplement: Supplementary file 1 — Figure S1 Figure S2 Figure S3 Figure S4 Figure S5 Figure S6 Figure S7 [file ACEL-21-e13692-s001.pdf]

Supplementary Figure 1

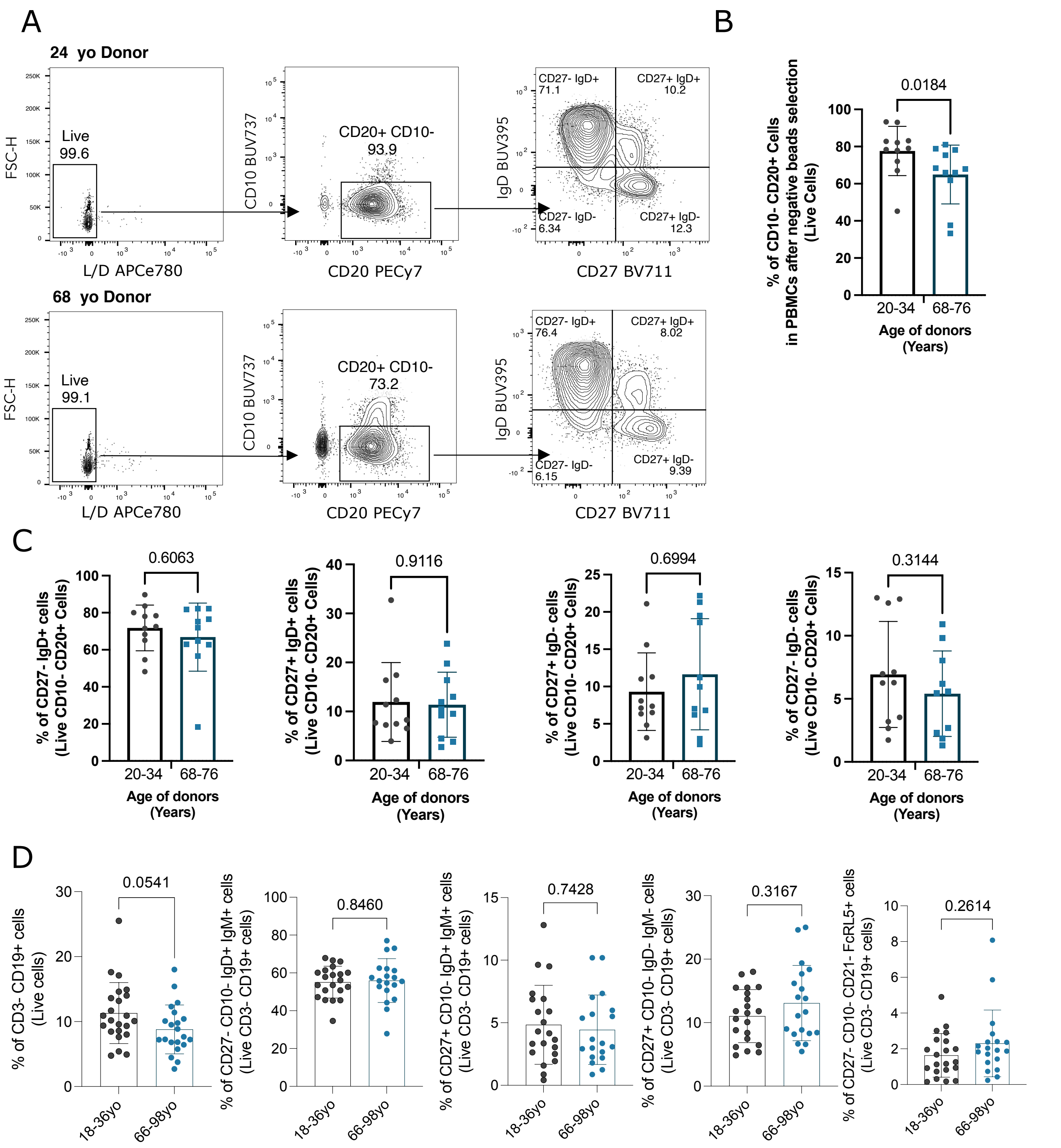

Supplementary Figure 1. Older humans have fewer B cells in their peripheral blood but similar proportions of naïve and memory cells, compared to young adult humans. A) Gating strategy for sorting naïve and memory B cells from PBMCs that have been pre-enriched for CD19+ cells using negative selection beads. Naïve B cells used in cultures were sorted live CD20+ CD10- CD27- IgD+ cells while memory B cells were sorted live CD27+IgD+/- and CD27- IgD- cells. B) Percentage of CD10- CD20+ cells out of live cells derived from PBMCs that have been pre-enriched for CD19+ cells, in young adult (20-34 years old) and older donors (68-76 years old). C) Percentage of naïve (CD27- IgD+) and the three subsets of memory cells (unswitched CD27+ IgD+, switched CD27+ IgD- and atypical CD27- IgD-) out of live CD10- CD20+ cells in young adult and older donors before stimulation. D) Dataset from a separate, larger independent cohort study, consisting of 21 young adult (18-36 years old) donors and 19 older (66-98 years old). From left to right: Percentage of CD3- CD19+ cells out of live cells, percentage of naïve (CD27- CD10- IgD+ IgM+) and the three subsets of memory cells (unswitched CD27+ CD10- IgD+IgM+, switched CD27+CD10- IgD- IgM- and atypical CD27- CD10-CD21- FcrL5+) out of live CD3- CD19+ cells in young adult and older donors before stimulation. Bar height corresponds to the mean, error bars indicate standard deviation, and each symbol represents values from independent donors. Statistics were calculated using Mann-Whitney U test. Data were representative of three independent repeat experiments.

Supplementary Figure 2

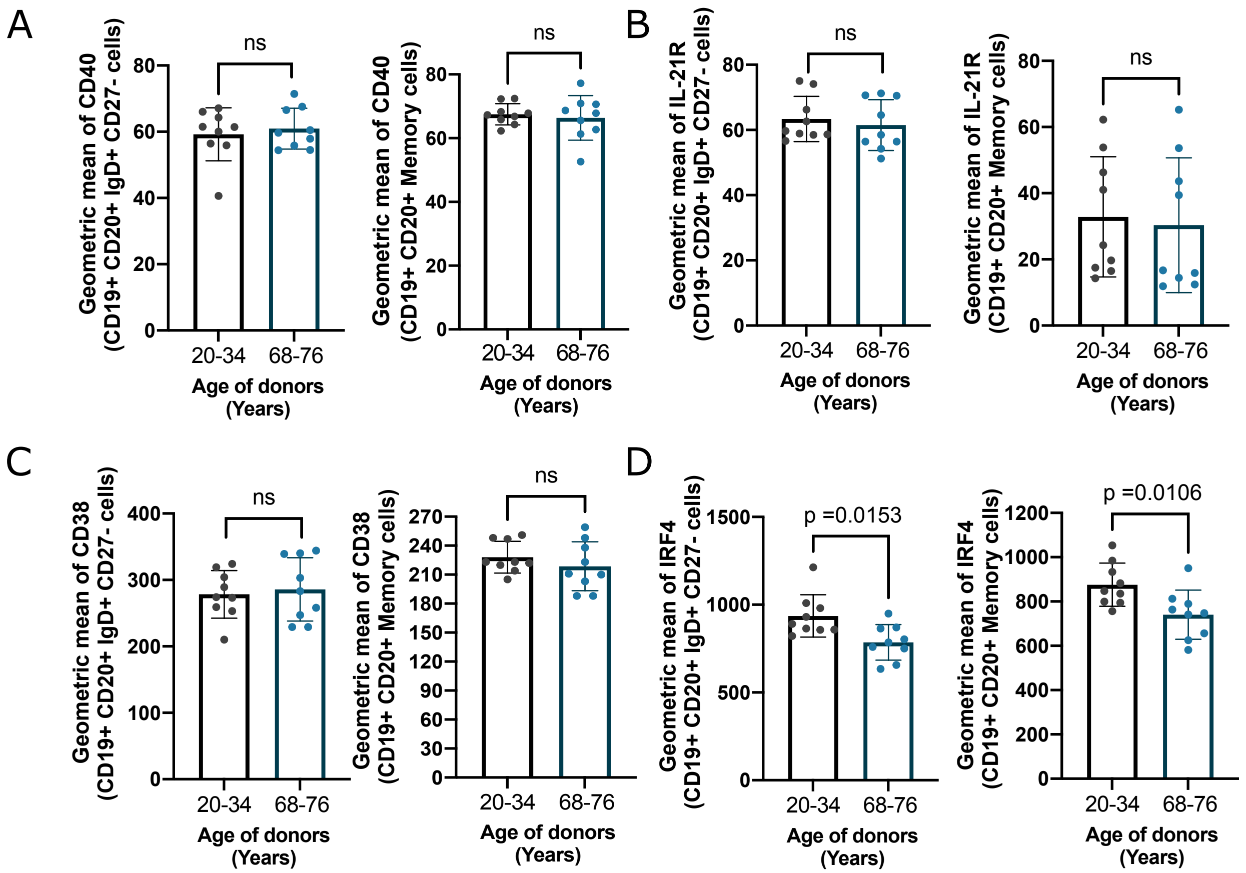

Supplementary Figure 2. Naïve and memory B cells from young and older donors have similar surface phenotypes before stimulation. Geometric mean of (A) CD40, (B) IL-21R, (C) CD38 and (D) IRF4 in naïve and memory B cells from young adult (20-34 years old) and older donors (68-76 years old). Bar height corresponds to the mean, error bars indicate standard deviation, and each symbol represents values from independent donors. Statistics were calculated using Mann-Whitney U test. Data were representative of two independent repeat experiments.

Supplementary Figure 3

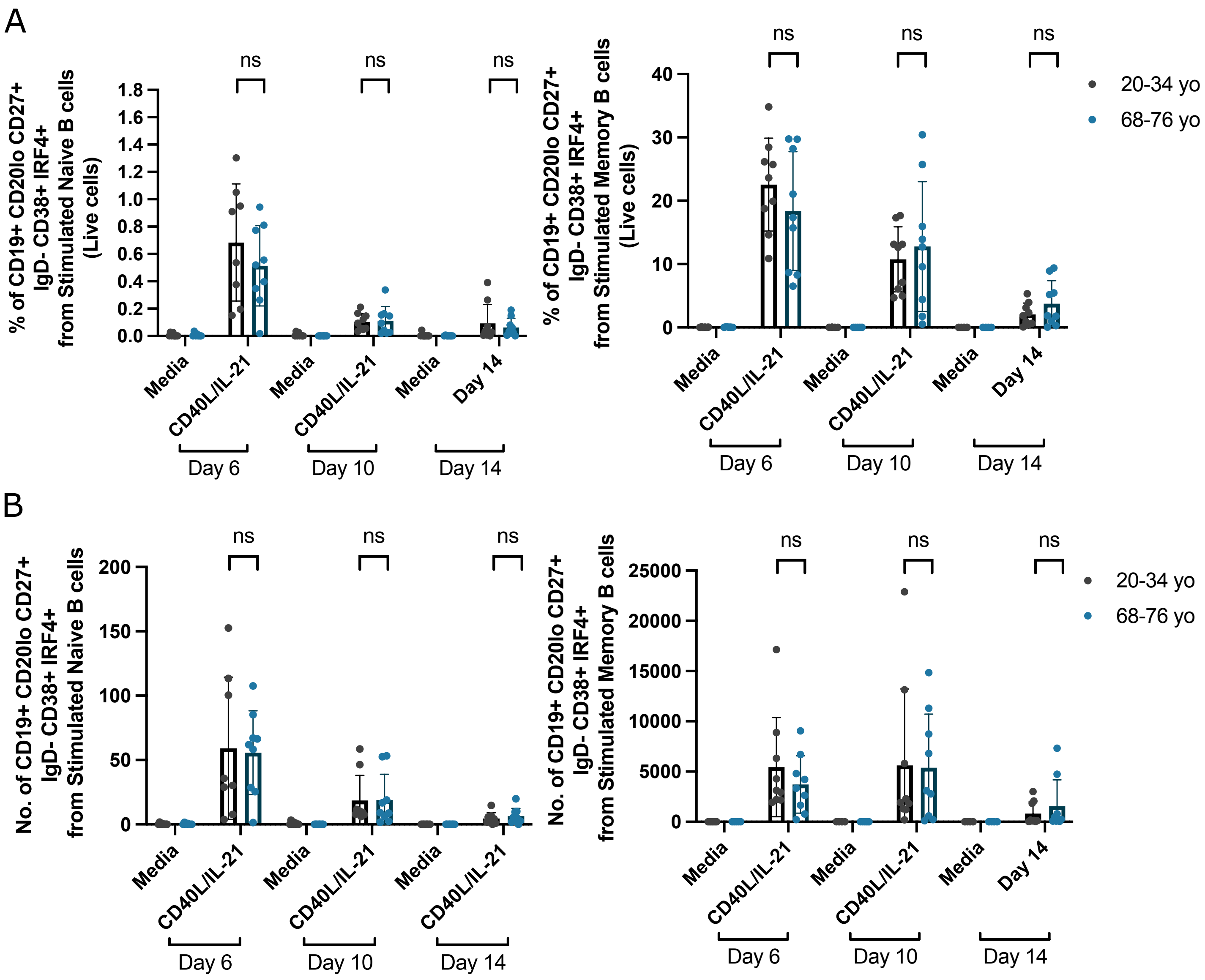

Supplementary Figure 3. Naïve and memory B cells from older humans do not have defects in plasma cell differentiation. A) Percentage and B) number of plasma cells out of live cells derived from naïve (left) and memory B cells (right) of young adult (20-34 years old) and older donors (68-76 years old) after 6, 10 and 14 days stimulation with CD40L and IL-21. Bar height corresponds to the mean, error bars indicate standard deviation, and each symbol represents values from independent donors. Statistics were calculated using Mann-Whitney U test. Data were representative of three independent repeat experiments.

Supplementary Figure 4

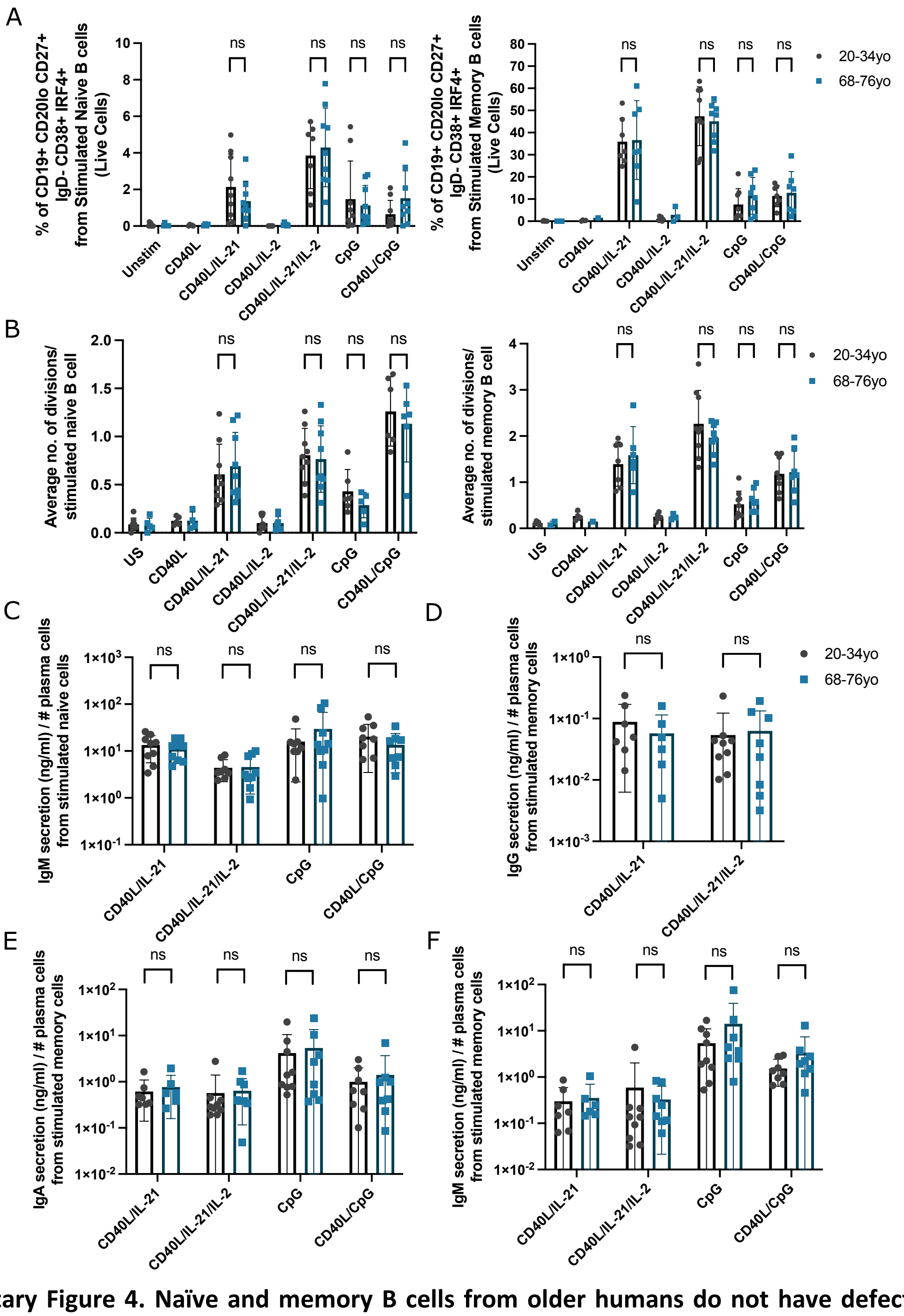

Supplementary Figure 4. Naïve and memory B cells from older humans do not have defects in plasma cell differentiation, proliferation and antibody secretion after stimulation with CD40L/IL-21/IL-2, CpG or CD40L/CpG. A) Percentage of plasma cells out of live cells derived from naïve (left) and memory B cells (right) of young adult (20-34 years old) and older donors (68-76 years old) after 6 days stimulation with a range of T-dependent and T-independent signals, namely CD40L, CD40L/IL-21, CD40L/IL-2, CD40L/IL-21/IL-2, CpG and CD40L/CpG. B) Average number of divisions per cell undergone by stimulated naïve B cells (left) and memory B cells (right) of young adult (20-34 years old) and older donors (68-76 years old) after 6 days stimulation with respective conditions. C-F) Graphs comparing the antibody-secreting capacity of plasma cells (Ig concentration divided by number of plasma cells) derived from naive (C) and memory cells (D-F) from younger and older donors after stimulation with respective T-dependent and T-independent stimulation conditions. Number of plasma cells was determined by flow cytometry with counting beads. Bar height corresponds to the mean, error bars indicate standard deviation, and each symbol represents values from independent donors. Statistics were calculated using Mann-Whitney U test. Data were representative of three independent repeat experiments.

Supplementary Figure 5

A

Panel 1

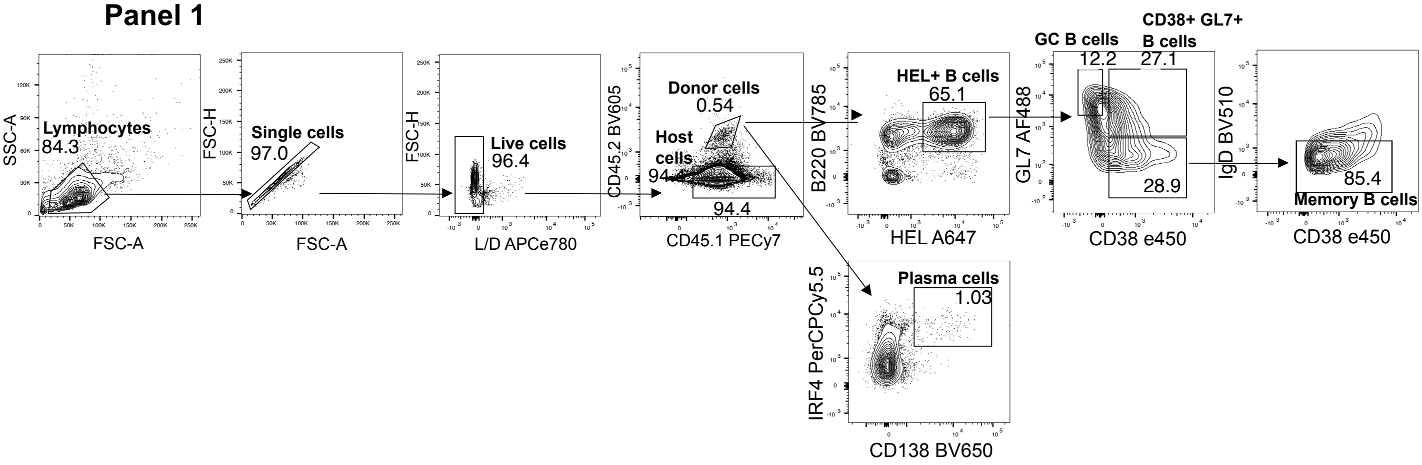

B

Panel 2

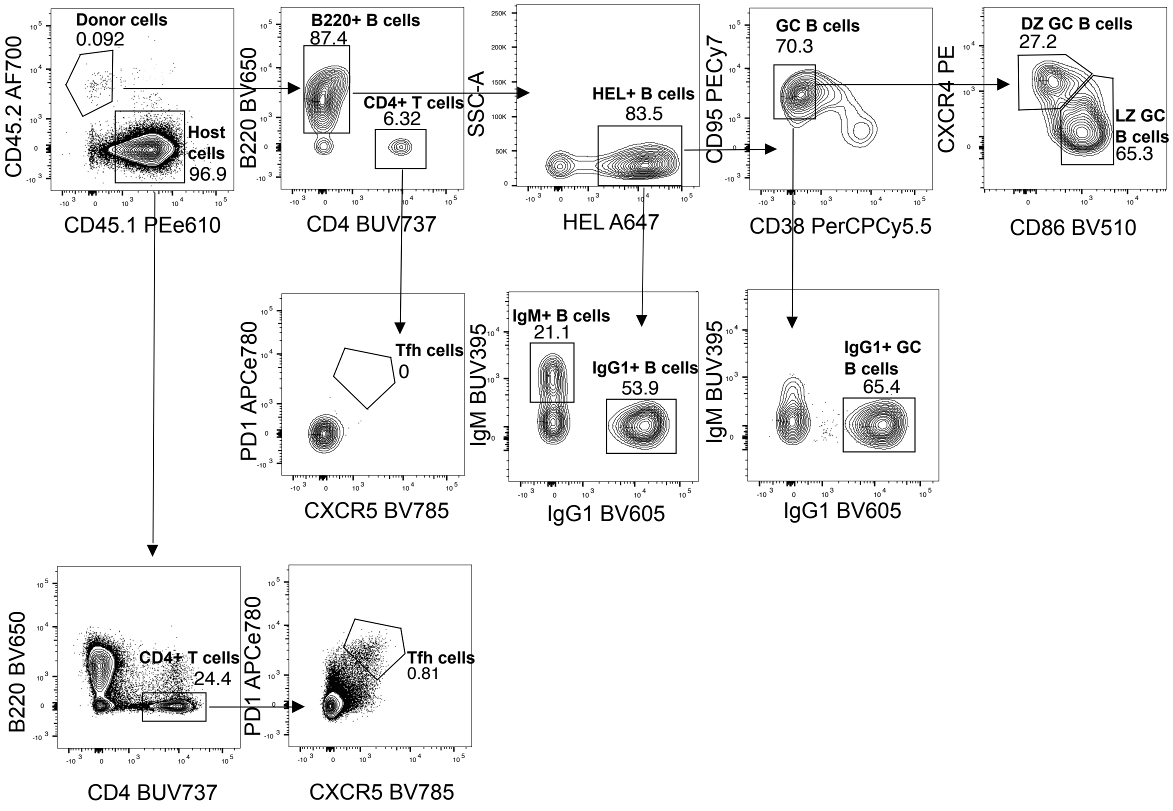

Supplementary Figure 5. Gating strategies for mouse *in vivo* transfer experiments for (A) Panel 1 and (B) Panel 2.

Supplementary Figure 6

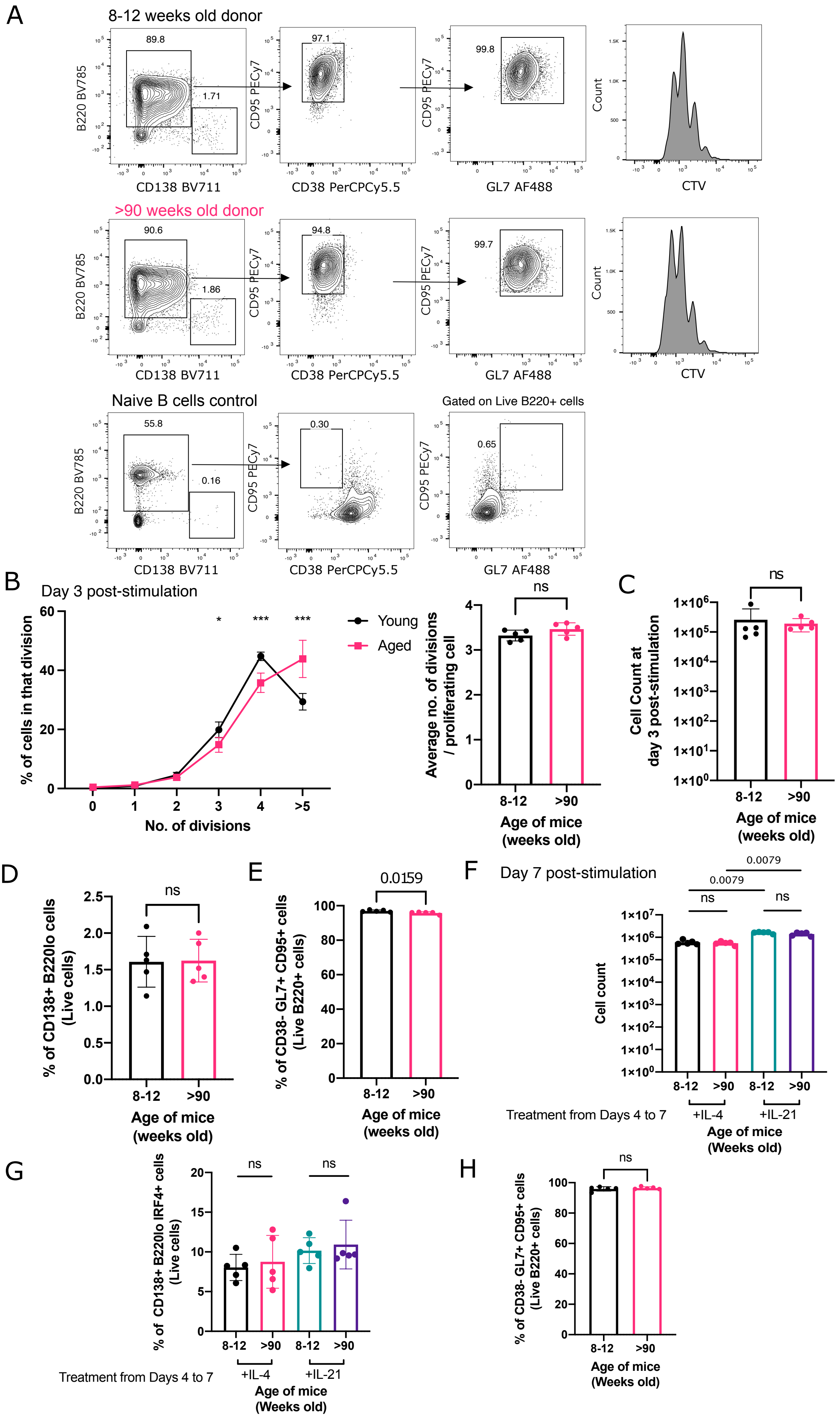

Supplementary Figure 6. Naïve follicular B cells from spleens of aged mice have no defects in proliferation and plasma cell differentiation when stimulated *in vitro* with the induced GC culture system. A) Representative flow cytometric plots showing gating strategies for CD138+ B220lo plasma cells and B220+ CD38- CD95+ GL7+ cells, as well as cell trace violet dilution, from stimulated naïve follicular B cells from 8-12 weeks old mice or aged >90-weeks old mice 3 days post-culture on 40LB cells with IL-4. Populations were pre-gated on live single lymphocytes and numbers adjacent to gates indicate percentage of parent population. Naïve B cells from an unimmunised mouse was included as a negative staining control. B) Graph showing the percentage of B cells in each division 3 days post-culture on 40LB cells with IL-4 (left) and graph showing the average number of divisions undergone per proliferating cell (right) by naïve follicular B cells from 8-12wo or >90wo mice. C) Cell counts of stimulated B cells at day 3 post-stimulation. 4 x 10^4 sorted naïve follicular cells were cultured in each well at day 0. D) Graph showing the percentage of CD138+ B220lo plasma cells out of live cells 3 days post-culture on 40LB cells with IL-4. E) Graph showing the percentage of CD38- GL7+ CD95+ cells out of live B220+ cells 3 days post-culture on 40LB cells with IL-4. F) Graph showing cell counts in cultures of stimulated naïve B cells from 8-12wo or >90 wo mice at day 7 post-culture on 40LB cells, with IL-4 or IL-21 added from days 4 to day 7. G) Graph showing the percentage of CD138+ B220lo IRF4+ cells in cultures of stimulated naïve B cells from 8-12wo or >90 wo mice at day 7 post-stimulation. H) Graph showing the percentage of CD38- GL7+ CD95+ cells in cultures of stimulated naïve B cells from 8-12wo or >90 wo mice at day 7 post-culture on 40LB cells and IL-4. Bar graphs show the results of one of two independent experiments (n = 5 per group/experiment). Bar height corresponds to the mean, error bars indicate standard deviation, and each symbol represents one biological replicate. Statistics were calculated using unpaired Mann-Whitney U test. For B), two-way ANOVA with Sidak's multiple comparisons test was used. Data were representative of two independent repeat experiments.

Supplementary Figure 7

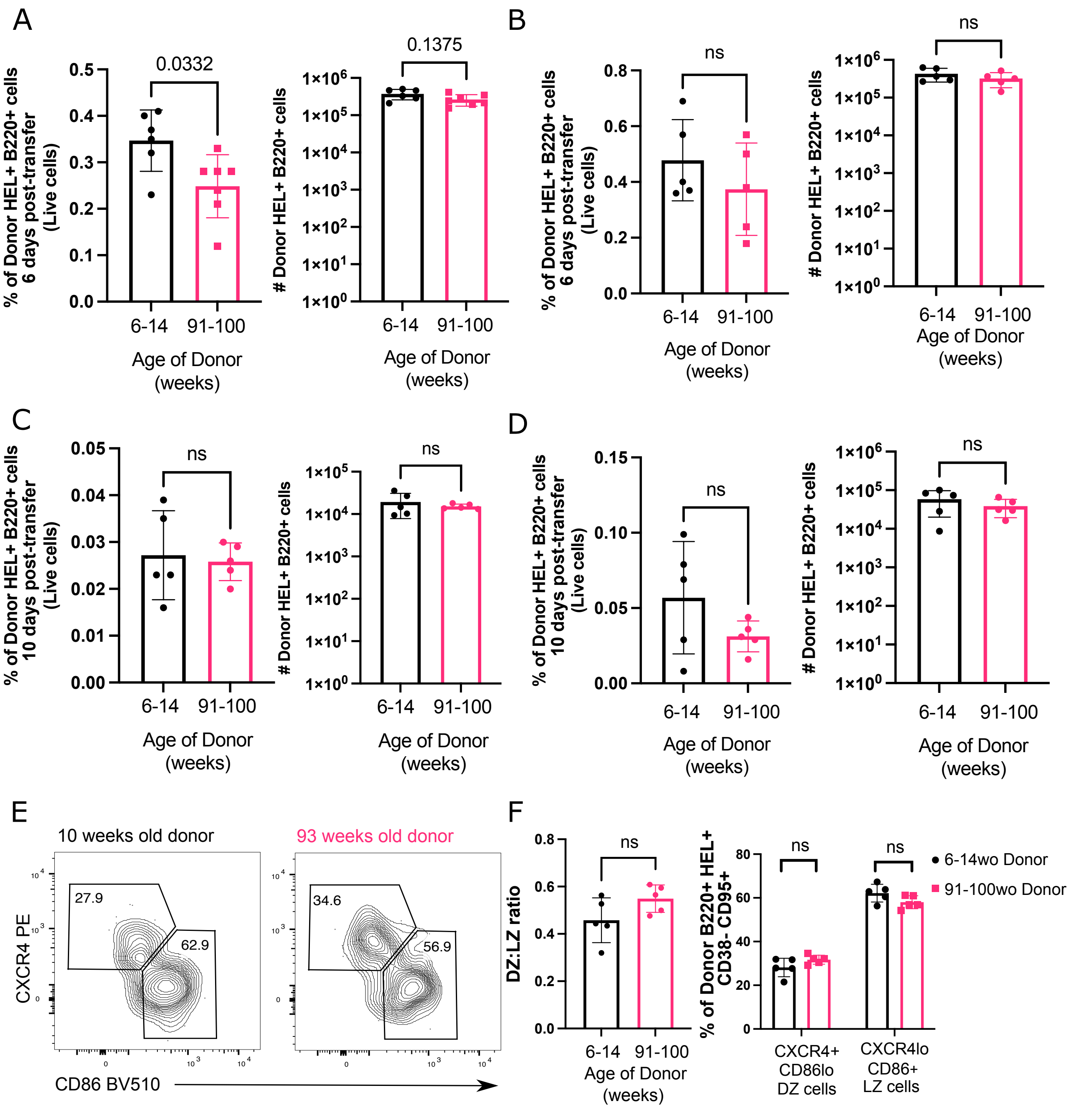

Supplementary Figure 7. B cells from aged donor mice do not have defects in homing to the spleen post-transfer and have no difference in DZ:LZ phenotype, compared to those from young donor mice. A-D) Percentage and number of transferred HEL-binding B cells derived from a young or aged donor mouse in recipient spleens (A-B) 6 days and (C-D) 10 days post-transfer and immunisation. E) Representative flow cytometric plots of dark zone (CXCR4+CD86lo) and light zone (CXCR4loCD86+) GC B cells. Numbers adjacent to gates indicate percentage of donor HEL+ B220+ CD38-CD95+ GC B cells. F) Graphs comparing dark zone: light zone (DZ:LZ) ratio among donor GC B cells derived from young and aged mice (left) and percentage of dark zone (CXCR4+ CD86lo) and light zone (CXCR4lo CD86+) cells among HEL+ donor-derived GC B cells (right). Bar height corresponds to the mean, error bars indicate standard deviation, and each symbol represents one biological replicate. Statistics were calculated using Mann-Whitney U test. Data were representative of two independent repeat experiments.
